# The relationship of baseline high-sensitivity C-reactive protein with incident cardiovascular events and all-cause mortality over 20 years

**DOI:** 10.1016/j.ebiom.2025.105786

**Published:** 2025-06-04

**Authors:** Adam Hartley, Somayeh Rostamian, Amit Kaura, Paris Chrysostomou, Paul Welsh, Cono Ariti, Naveed Sattar, Peter Sever, Ramzi Khamis

**Affiliations:** a National Heart and Lung Institute, Imperial College London, UK; b Institute of Cardiovascular and Medical Sciences, University of Glasgow, Glasgow, UK

**Keywords:** C-reactive protein, Cardiovascular events, Mortality, Inflammation

## Abstract

**Background:**

The prediction of future cardiovascular events in those with risk factors is important for the appropriate optimisation of preventative therapies for those at greatest risk. The value of high sensitivity C-reactive Protein (hsCRP) has been questioned in this regard. The objectives of this post-hoc analysis of a randomised controlled trial were to investigate the usefulness of baseline serum hsCRP for predicting very long-term cardiovascular events in patients with hypertension in the Anglo-Scandinavian Cardiac Outcomes Trial (ASCOT) Legacy Study.

**Methods:**

The ASCOT Legacy Study reports events up to 20 years of follow-up of the UK participants in the Lipid Lowering Arm of the original ASCOT trial. We examined outcomes related to serum hsCRP levels measured using a commercial ELISA, in tertiles or continuously, adjusting for classical cardiovascular risk factors as well as treatment allocation within ASCOT. The primary outcome was non-fatal myocardial infarction (MI) and fatal coronary heart disease (CHD); whilst secondary outcomes were all-cause mortality, total coronary events and procedures, total cardiovascular events and stroke.

**Findings:**

After excluding 3286 participants without hsCRP data, 5294 participants were included in the final cohort. The highest tertile of hsCRP was associated with the following outcomes compared to the lowest tertile: non-fatal myocardial infarction (MI) and fatal CHD (HR 1.32 [1.05–1.67]); total coronary events and procedures (HR 1.27 [1.09–1.47]); total cardiovascular events (HR 1.22 [1.08–1.37]); and all-cause mortality (HR 1.25 [1.10–1.42]). However, there was insufficient evidence regarding the association between hsCRP levels and stroke events. Addition of hsCRP in tertiles resulted in an improved net reclassification index for the prediction of non-fatal MI and fatal CHD at 20 years (9.68%, p < 0.0001).

**Interpretation:**

Higher baseline serum hsCRP levels can independently predict cardiovascular events and all-cause mortality at long-term follow-up in stable patients with hypertension.

**Funding:**

British Heart Foundation Clinical Research Fellowship (FS/17/16/32560), Wellcome Trust Clinical Research Fellowship (220572/Z/20/Z), and Sansour Fund at Imperial Healthcare Charity. The substudy of ASCOT Biomarker Programme was supported by Pfizer, New York, NY, USA. Infrastructure support was provided by the NIHR Imperial Biomedical Research Centre as well as the Imperial British Heart Foundation Research Excellence Award (4) (RE/24/130023).

**Research in context**

**Evidence before this study**

There is an evolving appreciation of the importance of inflammation in cardiovascular events, driven by emerging randomised data that demonstrate improved cardiovascular outcomes with immunomodulation therapies, such as Canakinumab or Colchicine. Circulating high sensitivity C-reactive protein (hsCRP) levels have been postulated as a useful tool to help predict future cardiovascular events. However, the importance of CRP in predicting very long-term outcomes has not yet been demonstrated.

**Added value of this study**

This large study, with over 5000 patients, benefits from long-term follow-up (up to 20 years) of patients with hypertension but without established cardiovascular disease. It demonstrates the significant legacy of even a minimally elevated hsCRP level at baseline (the highest tertile median was 6.41 mg/L, a value not widely considered to be clinically significant) in otherwise well individuals but with cardiovascular risk factors. When assessed in tertiles and continuously, higher hsCRP levels related to all adverse cardiovascular outcomes, after correction for potential confounders, with the exception of stroke and transient ischaemic attack. Indeed, the addition of hsCRP resulted in improved risk stratification for future myocardial infarctions.

**Implications of all the available evidence**

In this study, hsCRP aids in the identification of individuals at increased long-term cardiovascular risk, who may benefit from intensive risk reduction management. Moreover, this study adds supporting evidence for the role of low-level inflammation in predicting future cardiovascular events and suggests that hsCRP could be incorporated into clinical screening strategies for selecting patients for novel therapeutics, such as immunomodulatory treatments.

## Introduction

The importance of inflammation in atherogenesis and atherothrombosis has been greatly debated since it was first postulated some decades ago.^1^ Moreover, whilst there is a growing body of opinion that systemic inflammation may be a novel cardiovascular risk factor,^2^ reflected by the incorporation of various inflammatory autoimmune diseases into cardiovascular risk prediction algorithms,^3^ whether inflammation is causal in cardiovascular disease (CVD), or purely epiphenomenal, is still controversial. The measurement of plasma C-reactive protein (CRP), a hepatically-produced acute phase reactant, in relation to CVD, more recently with a highly sensitive immunoassay (hsCRP), has been at the centre of these debates.^4^

Initial interest in the role of CRP in CVD stemmed from early seminal studies that identified baseline CRP as an independent predictor of future myocardial infarction (MI) and stroke in healthy individuals.^5^^,^^6^ Moreover, modest anti-inflammatory therapy in the form of Aspirin was able to reduce the occurrence of myocardial infarction (MI) in those with the highest CRP levels.^5^ However, mendelian randomisation studies suggested that CRP itself was not causally related to CVD,^4^ unlike the interleukin-6 receptor, upstream of CRP in the inflammatory cascade.^7^ Nonetheless, the randomised placebo-controlled trial, Canakinumab Anti-inflammatory Thrombosis Outcome Study (CANTOS), used monoclonal antibody canakinumab targeted against IL-1β (upstream of IL-6) in patients with prior MI and raised hsCRP (>2 mg/L). Those in the therapeutic arm had a significant 15% reduction in recurrent CVD events over 3.7 years. This effect correlated with hsCRP reduction and was independent of lipid lowering.^8^ Critically, those that did not achieve on-treatment hsCRP <2 mg/L saw no therapeutic efficacy.^9^ Subsequent studies have confirmed the role of other immune modulating drugs in treating coronary artery disease,^10^^,^^11^ which is now reflected in international guidelines, where the use of Colchicine, a broad-spectrum anti-inflammatory agent, is recommended for cardiovascular risk reduction following the successful COLchicine Cardiovascular Outcomes Trial (COLCOT)^11^ and LOw-DOse COlchicine (LoDoCo-2)^10^ studies.^12^

Thus, gaining an assessment of a patient's inflammatory state may be helpful in the management of CVD in the clinical setting, regardless of whether the inflammation is arising systemically due to some other cause, or more directly from vascular endothelium. Accordingly, we have recently shown a positive and graded relationship between mildly elevated hsCRP levels, measured at presentation in those with suspected MI, and all-cause mortality at 3-years, which was independent of troponin levels.^13^

In the present study we sought to address a further unanswered question: whether hsCRP levels measured at baseline in a stable population but with significant cardiovascular risk factors could predict very long-term cardiovascular outcomes. This was possible through analysis of the Lipid Lowering Arm (LLA) of the Anglo-Scandinavian Cardiac Outcomes Trial (ASCOT) Legacy study. The ASCOT study was a multicentre randomised trial, randomising patients with hypertension into Amlodipine-based or Atenolol-based blood pressure-lowering (BPL) treatment. The LLA substudy took patients with non-fasting total cholesterol concentrations ≤6.5 mmol/L and randomly assigned them to an additional atorvastatin 10 mg or placebo. The ASCOT Legacy study recently reported 20 year stroke outcomes after long-term follow-up of the UK participants in the original ASCOT-LLA study.^14^

## Methods

### Study design and participants

The detailed ASCOT protocol, including study design, organisation, clinical measurements and endpoint definitions, has been published previously (NCT01499511).^15^ Briefly, the ASCOT was a 2 × 2 factorial study that recruited patients in the United Kingdom (UK), Ireland and Nordic countries between 1998 and 2002. Patients aged 40–79 were eligible for participation in the blood pressure-lowering arm (BPLA) if they had either untreated hypertension (systolic blood pressure of 160 mmHg or more, diastolic blood pressure of 100 mmHg or more, or both) or treated hypertension (systolic blood pressure of 140 mmHg or more, diastolic blood pressure 90 mmHg or more, or both) with three additional cardiovascular risk factors including male sex, age over 55 years old, smoking, left-ventricular hypertrophy (detected by electrocardiogram or echocardiogram); other specified abnormalities on electrocardiogram, type 2 diabetes; peripheral arterial disease; previous stroke or transient ischaemic attack (TIA); microalbuminuria or proteinuria; ratio of plasma total cholesterol to HDL-cholesterol of six or higher; or family history of premature CHD. Individuals with previous MI; currently treated angina; a cerebrovascular event within the previous three months; fasting triglycerides higher than 4.5 mmol/L; heart failure; uncontrolled arrhythmias; or any clinically important haematological or biochemical abnormality on routine screening were excluded from the study. Sex data were self-reported by study participants.

Participants in ASCOT-BPLA were randomly assigned to either amlodipine 5–10 mg adding perindopril 4–8 mg as required (amlodipine-based regimen; n = 9639) or atenolol 50–100 mg adding bendroflumethiazide 1.25–2.5 mg and potassium as required (atenolol-based regimen; n = 9618). In addition, patients with a non-fasting total cholesterol of 6.5 mmol/L or less, currently untreated with a statin or fibrate, and whose physicians did not intend to treat them with a statin or fibrate, were recruited in Lipid Lowering Arm (LLA), and were randomly assigned to atorvastatin 10 mg daily (n = 5168) or placebo (n = 5137). The ASCOT-LLA was stopped prematurely after a median of 3.3 (IQR 2.7–3.7) years of active follow-up because of substantial benefit in those assigned atorvastatin. Trial physicians were invited to offer atorvastatin to all ASCOT-LLA patients until the end of ASCOT-BPLA.^16^

### Ethics

All study participants gave written informed consent for inclusion in the study, and the investigation conformed to the principles outlined in the Declaration of Helsinki. The investigators obtained approval from the South East Scotland Research Ethics Committee (18/SS/0016), the Health Research Authority Confidentiality Advisory Group (18/CAG/0044), the Independent Group Advising on the Release of Data of NHS Digital, as well as the Public Benefit and Privacy Panel for Health and Social Care of NHS Scotland.

### Measurement of hsCRP and lipids

Serum samples for CRP analysis were collected at baseline and after 6-months, and were stored at −80 °C. High-sensitivity CRP concentrations were measured using the ARCHITECT hsCRP assay (Abbott Laboratories, Abbott Park, IL, USA), a chemiluminescent microparticle immunoassay. This was performed according to the manufacturer's instructions by technicians blinded to the case–control status of the samples. The lower limit of sensitivity was 0.1 mg/L and the coefficient of variation was <4%. Fasting lipids were reported at the baseline of the study and then regularly measured during the trial. Fasting lipid levels were measured at baseline, followed by a six-month assessment, and then annually until the end of the study.

### ASCOT legacy cohort

The ASCOT Legacy cohort consisted of all 8580 ASCOT trial participants from the UK (England, Scotland and Wales). These patients were followed up until the end of the trial, during which period 717 died and the rest were flagged with the Office for National Statistics and the General Register Office for Scotland for post-trial follow-up. In this report, we included 5294 patients with available data on hsCRP at baseline up to 31st January 2019.

Clinical outcomes collected from electronic health records were non-fatal MI or fatal coronary heart disease (CHD); fatal or non-fatal stroke or TIA; total coronary events and procedures (comprising all MI as well as revascularisation procedures [percutaneous coronary intervention or coronary artery bypass grafting]); total cardiovascular events (a composite of all above outcomes) and all-cause mortality. The median follow-up time was 14.20 (interquartile range [IQR] [0.01–20.93]) years.

### Outcomes

The prespecified primary outcome was the relationship of hsCRP with non-fatal or fatal CHD, the primary outcome of the original ASCOT.^17^ Secondary outcomes were non-fatal and fatal stroke/TIA, total coronary events and procedures, total cardiovascular events as well as all-cause mortality.

### Statistics

All UK participants in the ASCOT-LLA with hsCRP and total cholesterol levels measured at baseline were included in the final analysis. Where linkage was not possible (largely because they had not consented to long-term mortality follow-up), we censored participants at the end of trial follow-up period.

Cox proportional hazards regression analysis was used to perform survival analysis, reporting hazard ratios (HR) with 95% confidence intervals (CI) and associated p-values. Using Martingale residuals, nonlinearity was detected in the relationship between the log hazard and hsCRP. To model nonlinear relationships, we treated hsCRP as a categorical and continuous variable. The proportional hazards assumption of the Cox regression model was satisfied across tertiles, and subgroups based on CRP levels (hsCRP <2 mg/L, hsCRP 2–4.9 mg/L, and hsCRP ≥5 mg/L). We also used restricted cubic splines for Cox analyses to model the hazard ratio across different levels of hsCRP. Preliminary analyses suggested that 3 unforced knots should be used to model hsCRP level in the restricted cubic spline analyses. A higher number of knots was not chosen to prevent overfitting, which could compromise the model's generalisability. The placement of the three knots for restricted cubic splines is at the 25th, 50th, and 75th percentiles of the predictor variable, a standard approach that ensures the spline captures the relationship between the predictor and outcome variable while further avoiding overfitting. This method is flexible enough to account for changes in the central distribution of the data.

Two-sided p-values <0.05 were considered statistically significant. Data were reported as mean with standard deviation or median with IQR as appropriate.

Analyses are reported in an unadjusted fashion (Crude Model); Model 1 which was adjusted for age, sex, socio-economic status (years of education) and ethnicity; Model 2 which further adjusted for current smoking status (at baseline), body mass index, baseline systolic blood pressure, creatinine, diabetes, history of vascular diseases (coronary, cerebral, peripheral), antihypertensive medication, and allocation to the BP lowering and LL lowering strategies. Model 3 was designed for use when the level of cholesterol and hsCRP were to be considered, in which Model 2 was further adjusted for baseline hsCRP and total cholesterol, to demonstrate that correlation was independent of the baseline measurements. The confounders were selected based on the research question, biological mechanisms, and prior literature to establish that they are associated with both the exposure and the outcome without being on the causal pathway. They were measured at baseline, prior to the exposure, which helped reduce the risk of mistakenly adjusting for mediators.^18^ Alongside baseline hsCRP measurements, we also explored the relationships with baseline total cholesterol, temporal changes in hsCRP or total cholesterol at 6-months (in those with available data), and with follow up that ended when the trial finished, rather than long-term follow-up. Moreover, in supplementary analyses, we assessed the impact of ASCOT treatment allocation on these relationships by including only participants who were randomised to atorvastatin or placebo, excluding non-randomised individuals from the analysis (Supplementary Tables S1, S2 and S3). To assess the additive value of hsCRP to traditional cardiovascular risk factors, we compared two models: Model A included all the variables included in Model 1 and 2, as outlined above. Model B included all variables in Model A plus hsCRP, as either a categorical variable (in tertiles), or continuous variable. Cox proportional hazards models were used to assess the associations, and Kaplan–Meier curves with life tables were generated to evaluate the impact of hsCRP as a novel marker. p-values were calculated using the log-rank test (Mantel–Cox test), which evaluates the null hypothesis that there is no difference in cumulative hazard rates between the predefined hsCRP groups over the 20 year follow-up period. The test compares the observed versus expected number of events at each time point under the assumption of identical hazard functions across groups. A significant p-value (p < 0.05) indicates that at least one group's cumulative hazard curve diverges significantly from the others.

To conduct sensitivity analyses, we repeated the analysis using competing risks regression models, which considered non-cardiovascular mortality competing risks to events.

The likelihood ratio (LR) test was used to provide a formal statistical framework to compare the goodness of fit of the two competing models. Specifically, it tests whether the more complex model, Model B, significantly improves the fit to the data compared to the simpler model, Model A. Discrimination was assessed by comparing the area under the receiver operating characteristic curves (AUROCs) using the DeLong nonparametric approach. Even if changes in AUROCs are statistically significant, they may not necessarily translate to clinically meaningful improvements in risk prediction. We therefore considered measures of improvement in risk classification or reclassification, which are essential for assessing the clinical utility of adding new risk markers. We used Net Reclassification Improvement (NRI) and Integrated Discrimination Improvement (IDI) for evaluating risk reclassification. NRI quantifies how effectively individuals are reclassified into more appropriate risk categories by the new model compared to a baseline model. IDI measures the overall improvement in the model's ability to distinguish between individuals who do and do not experience the event, without relying on predefined risk categories. Further categorical NRI analyses for hsCRP were undertaken to test reclassification with categories (<10%, 10–20% and >20% risk) selected to define as low, intermediate and high risk for all outcomes. A Sankey diagram has been used as a visual tool to illustrate the flow of patients between risk categories (high, intermediate, and low) when hsCRP is incorporated into the risk prediction model.

**Fig. 1:**
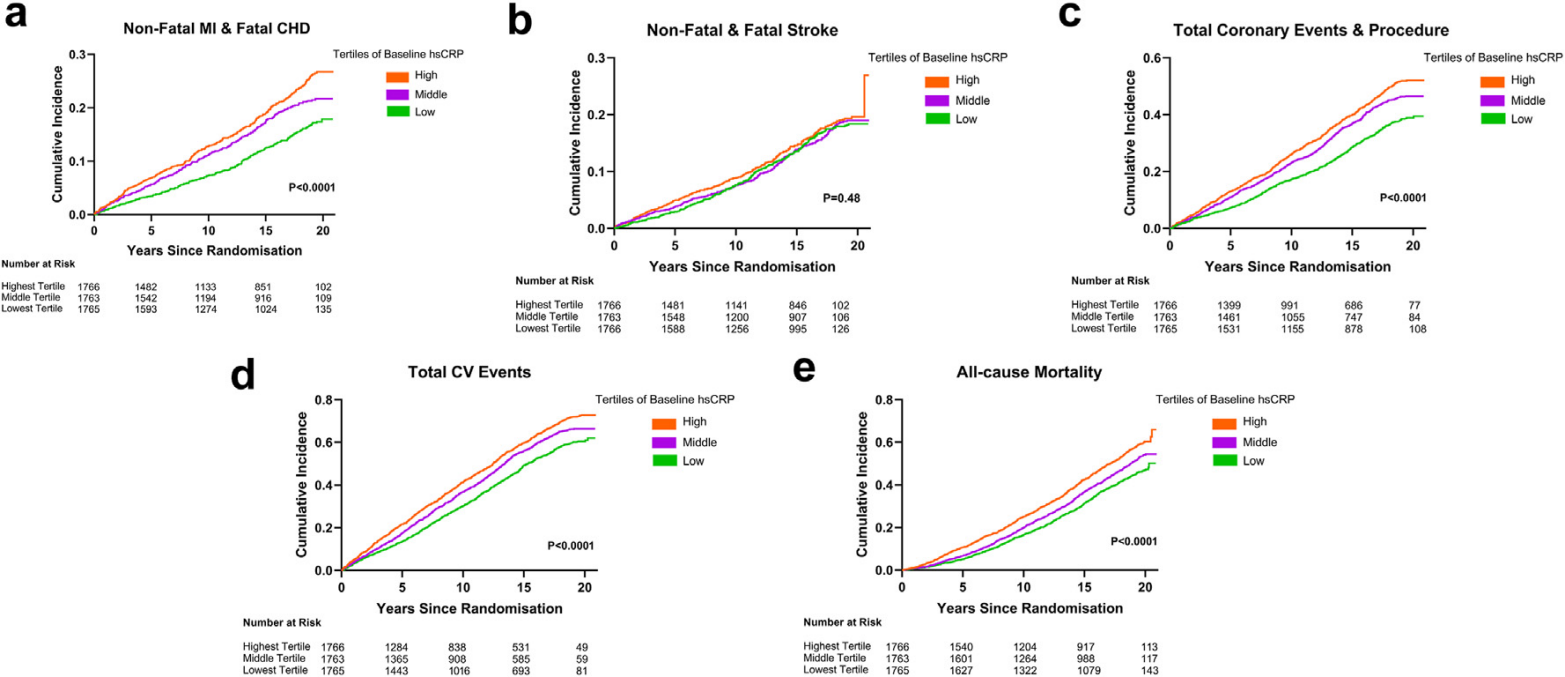
**Kaplan Meier curves in relation to baseline hsCRP and outcomes over 20 year follow-up in the ASCOT Legacy cohort (n = 5294)**. (a)—Non-fatal MI and fatal CHD. (b)—Non-fatal and fatal stroke. (c)—Total coronary events and procedures (d)—Total cardiovascular events. (e)—All-cause mortality. CHD: coronary heart disease; CV: cardiovascular; hsCRP: high sensitivity C-reactive protein. Kaplan Meier curves were generated using GraphPad Prism 9 (GraphPad Software, La Jolla, CA, USA). [p-values reflect overall differences in survival probability between all hsCRP groups via the log-rank test (two-sided, α = 0.05). The test evaluates whether the time-to-event distributions differ significantly across groups during follow-up.] [Life tables provide a detailed summary of events and survival data, showing the probability and the number of individuals at risk over specified intervals. It is displayed beneath the Kaplan–Meier curves and indicates the number of participants at risk at each time point, provides context for interpreting the curve (particularly as the sample size decreases over time), and helps to assess the reliability of survival estimates.].

All analyses up to outcome risk prediction with hsCRP were performed using Stata 14 (STATA Corporation, College Station, TX, USA), with R version 3.5.0 (R Core Team, Vienna, Austria) used for subsequent analyses and graphs were generated using GraphPad Prism 9 (La Jolla, CA, USA). Table 1, Table 2, Table 3, Table 4, Table 5 and Supplementary Tables S1 to S3 were produced using Stata 14 (StataCorp, College Station, TX, USA). Fig. 1 and Supplementary Figure S2 were generated using GraphPad Prism 9 (GraphPad Software, La Jolla, CA, USA). Table 6, Supplementary Table S4, and Supplementary Figures S1 and S3 were produced using R version 3.5.0 (R Core Team, Vienna, Austria).

**Table 1:** Baseline characteristics of the study population in the LL Arm of ASCOT Legacy (n = 5294).

Values	Not randomised (n = 805)	Placebo (n = 2232)	Atorvastatin (n = 2257)
Age (years), mean (SD)	64.7 (7.8)	64.5 (8.2)	64.5 (8.1)
Male, n (%)	639 (79.4)	1951 (87.4)	1962 (86.9)
Female, n (%)	166 (20.6)	281 (12.6)	295 (13.1)
Education (year), n (%)^a^			
Left school at or before age 16	659 (81.9)	1762 (78.9)	1741 (77.1)
Left school after age 17	146 (18.1)	470 (21.1)	516 (22.9)
Race, White (%)	805 (100.0)	1964 (88.0)	1989 (88.1)
Current Smokers, n (%)	185 (23.0)	572 (25.6)	595 (26.4)
BMI (kg/m^2^), mean (SD)	29.1 (4.3)	28.8 (4.6)	28.8 (4.9)
SBP (mmHg), mean (SD)	162.2 (17.7)	162.3 (17.8)	162.2 (17.3)
DBP (mmHg), mean (SD)	92.4 (10.6)	92.7 (9.7)	92.4 (9.9)
Creatinine (mmol/l), mean (SD)	100.7 (17.0)	100.8 (17.3)	100.6 (17.0)
Total Cholesterol (mmol/l), mean (SD)	6.5 (1.1)	5.4 (0.8)	5.4 (0.8)
LDL (mmol/l), mean (SD)^a^	4.3 (1.0)	3.5 (0.8)	3.4 (0.7)
Diabetes, n (%)	196 (24.3)	626 (28.0)	616 (27.3)
Peripheral Vascular Disease, n (%)	0	149 (6.7)	158 (7.0)
Coronary Artery Disease, n (%)	160 (19.9)	379 (17.0)	336 (14.9)
Stoke and TIA, n (%)	0	235 (10.5)	227 (10.1)
Antihypertensive medication, n (%)	720 (89.4)	2054 (92.0)	2060 (91.3)
Allocated to BPL medication, n (%)			
Amlodipine	404 (49.8)	1126 (50.4)	1117 (49.5)
Atenolol	401 (49.8)	1106 (49.6)	1140 (50.5)

**Abbreviations:** n: number, CV: cardiovascular, SD: standard deviation, Y: year, BMI: body mass index, kg/m2: kilogram per square meters, SBP: systolic blood pressure, mmHg: millimetre of mercury, DBP: diastolic blood pressure, LDL: low-density lipoprotein, TIA: transient ischemic attack, BPL: Blood Pressure Lowering.

a 3 subjects had missing data on education, and 310 subjects had missing data on LDL cholesterol, although they have a complete total cholesterol profile.

**Table 2:** Association between tertile of baseline hsCRP and outcomes in the ASCOT Legacy cohort (n = 5294).

Outcomes	Events (%)	Rate^a^	Crude, HR (95% CI)	Model 1, HR (95% CI)	Model 2, HR (95% CI)
**Non-fatal MI & fatal CHD**					
Lowest Tertile	232 (13.1)	9.18	1.00 (Ref)	1.00 (Ref)	1.00 (Ref)
Middle Tertile	297 (16.8)	12.40	1.36 (1.14–1.61)	1.30 (1.10–1.55)	1.20 (0.96–1.51)
Highest Tertile	343 (19.4)	15.00	1.65 (1.39–1.95)	1.63 (1.37–1.92)	1.32 (1.05–1.67)
p-value			*<0.001*	*<0.001*	*0.02*
**Non-fatal & fatal stroke**					
Lowest Tertile	244 (13.8)	9.78	1.00 (Ref)	1.00 (Ref)	1.00 (Ref)
Middle Tertile	240 (13.6)	10.04	1.03 (0.86–1.23)	0.96 (0.80–1.14)	0.90 (0.72–1.13)
Highest Tertile	246 (13.9)	10.75	1.11 (0.93–1.33)	1.02 (0.85–1.22)	1.02 (0.81–1.29)
p-value			*0.24*	*0.83*	*0.89*
**Total coronary events & procedure**					
Lowest Tertile	536 (30.4)	22.97	1.00 (Ref)	1.00 (Ref)	1.00 (Ref)
Middle Tertile	650 (36.9)	29.98	1.32 (1.18–1.48)	1.27 (1.13–1.42)	1.22 (1.05–1.41)
Highest Tertile	711 (40.3)	34.55	1.54 (1.38–1.72)	1.49 (1.33–1.67)	1.27 (1.09–1.47)
p-value			*<0.001*	*<0.001*	*0.01*
**Total CV events**					
Lowest Tertile	902 (51.1)	43.05	1.00 (Ref)	1.00 (Ref)	1.00 (Ref)
Middle Tertile	992 (56.3)	51.49	1.21 (1.11–1.33)	1.15 (1.05–1.26)	1.09 (0.97–1.23)
Highest Tertile	1082 (61.3)	59.79	1.42 (1.30–1.55)	1.37 (1.25–1.49)	1.22 (1.08–1.37)
p-value			*<0.001*	*<0.001*	*0.01*
**All-cause mortality**					
Lowest Tertile	733 (41.5)	28.08	1.00 (Ref)	1.00 (Ref)	1.00 (Ref)
Middle Tertile	834 (47.3)	33.26	1.21 (1.09–1.33)	1.11 (1.00–1.22)	1.01 (0.89–1.15)
Highest Tertile	957 (54.2)	39.87	1.47 (1.33–1.61)	1.38 (1.27–1.53)	1.25 (1.10–1.42)
p-value			*<0.001*	*<0.001*	*<0.001*

**Abbreviations:** HR: Hazard Ratio, CI: Confidence Interval CV: Cardiovascular, MI: Myocardial Infarction, CHD: Coronary Heart Diseases. hsCRP; High sensitivity C-reactive protein; Ref; Reference.

Reference (Ref) refers to the baseline group in a comparison, against which all other groups are evaluated when calculating the Hazard Ratio (HR).

**Model 1:** Adjusted for age, sex, socio-economic status (years of education) and ethnicity. **Model 2:** Model 1 adjusted further for a current smoker, body mass index, baseline SBP, creatinine, total cholesterol, diabetes, history of vascular diseases (coronary, cerebral, peripheral), history of antihypertensive medication, and allocation to blood pressure-lowering and lipid-lowering.

hsCRP categories: Lowest Tertile: n = 1765 à hsCRP: 0.01–1.51 [Median (IQR): 0.88 (0.57–1.21)]. Middle Tertile: n = 1763 à hsCRP: 1.52–3.71 [Median (IQR): 2.41 (1.94–2.98)]. Highest Tertile: n = 1766 à hsCRP: 3.72–191.37 [Median (IQR): 6.41 (4.81–10.44)].

Cox proportional hazards regression analysis was used to perform survival analysis, reporting hazard ratios (HR) with 95% confidence intervals (CI) and associated p-values.

a Per 1000 person-years.

**Table 3:** Association between baseline hsCRP in subgroups and outcomes in the ASCOT Legacy cohort (n = 5294).

Outcomes	Events (%)	Rate^a^	Crude, HR (95% CI)	Model 1, HR (95% CI)	Model 2, HR (95% CI)
**Non-fatal MI & fatal CHD**					
hsCRP <2	315 (14.0)	9.84	1.00 (Ref)	1.00 (Ref)	1.00 (Ref)
hsCRP 2 to 4.9	313 (17.6)	12.96	1.32 (1.13–1.55)	1.29 (1.10–1.51)	1.24 (1.00–1.53)
hsCRP ≥5	244 (19.4)	15.95	1.57 (1.33–1.85)	1.56 (1.32–1.85)	1.22 (0.96–1.57)
p-value			*<0.001*	*<0.001*	*0.07*
**Non-fatal & fatal Stroke**					
hsCRP <2	310 (13.7)	9.81	1.00 (Ref)	1.00 (Ref)	1.00 (Ref)
hsCRP 2 to 4.9	235 (13.2)	9.73	1.00 (0.84–1.18)	0.95 (0.80–1.13)	0.94 (0.75–1.16)
hsCRP ≥5	185 (14.7)	11.59	1.20 (1.00–1.44)	1.12 (0.93–1.34)	1.07 (0.84–1.36)
p-value			*0.08*	*0.34*	*0.70*
**Total coronary events & procedure**					
hsCRP <2	716 (31.8)	24.27	1.00 (Ref)	1.00 (Ref)	1.00 (Ref)
hsCRP 2 to 4.9	667 (37.4)	30.65	1.28 (1.15–1.42)	1.24 (1.12–1.38)	1.12 (0.97–1.28)
hsCRP ≥5	514 (40.9)	35.85	1.51 (1.35–1.69)	1.48 (1.32–1.66)	1.29 (1.10–1.51)
p-value			*<0.001*	*<0.001*	*0.01*
**Total CV events**					
hsCRP <2	1179 (52.3)	44.49	1.00 (Ref)	1.00 (Ref)	1.00 (Ref)
hsCRP 2 to 4.9	1018 (57.1)	52.68	1.20 (1.11–1.31)	1.17 (1.08–1.28)	1.09 (0.98–1.22)
hsCRP ≥5	779 (62.0)	62.35	1.44 (1.31–1.57)	1.40 (1.28–1.53)	1.26 (1.11–1.42)
p-value			*<0.001*	*<0.001*	*<0.001*
**All-cause mortality**					
hsCRP <2	950 (42.1)	28.68	1.00 (Ref)	1.00 (Ref)	1.00 (Ref)
hsCRP 2 to 4.9	874 (49.0)	34.60	1.23 (1.12–1.35)	1.16 (1.06–1.27)	1.07 (0.95–1.20)
hsCRP ≥5	700 (55.7)	41.68	1.51 (1.37–1.66)	1.46 (1.32–1.61)	1.37 (1.20–1.55)
p-value			*<0.001*	*<0.001*	*<0.001*

**Abbreviations:** HR: Hazard Ratio, CI: Confidence Interval CV: Cardiovascular, MI: Myocardial Infarction, CHD: Coronary Heart Diseases. hsCRP; High sensitivity C-reactive protein; Ref; Reference.

Reference (Ref) refers to the baseline group in a comparison, against which all other groups are evaluated when calculating the Hazard Ratio (HR).

**Model 1:** Adjusted for age, sex, socio-economic status (years of education) and ethnicity. **Model 2:** Model 1 adjusted further for a current smoker, body mass index, baseline SBP, creatinine, total cholesterol, diabetes, history of vascular diseases (coronary, cerebral, peripheral), history of antihypertensive medication, and allocation to blood pressure-lowering and lipid-lowering.

hsCRP categories: hsCRP <2 mg/L: n = 2255 à [Median (IQR): 1.06 (0.65–1.46)]. hsCRP 2–4.9 mg/L: n = 1783 à [Median (IQR): 3.07 (2.47–3.84)]. hsCRP ≥5 mg/L: n = 1256 à [Median (IQR): 8.15 (6.12–13.23)].

Cox proportional hazards regression analysis was used to perform survival analysis, reporting hazard ratios (HR) with 95% confidence intervals (CI) and associated p-values.

a Per 1000 person-years.

**Table 4:** Risk of long-term cardiovascular events and mortality in relation to baseline hsCRP and total Cholesterol (n = 5294).

Outcomes	Events (%)	Rate^a^	Crude, HR (95% CI)	Model 1, HR (95% CI)	Model 2, HR (95% CI)
**Non-fatal MI & fatal CHD**					
hsCRP<2 and Total Chol <5	85 (13.2)	9.46	1.00 (Ref)	1.00 (Ref)	1.00 (Ref)
hsCRP<2 and Total Chol ≥5	231 (14.3)	9.99	1.05 (0.82–1.35)	1.07 (0.83–1.37)	1.01 (0.74–1.38)
hsCRP ≥2 and Total Chol <5	114 (15.9)	12.38	1.32 (0.99–1.75)	1.30 (0.98–1.72)	1.17 (0.83–1.66)
hsCRP ≥2 and Total Chol ≥5	442 (19.1)	14.36	1.52 (1.21–1.92)	1.52 (1.20–1.91)	1.27 (0.95–1.71)
p-value			*<0.001*	*<0.001*	*0.03*
**Non-fatal & fatal Stroke**					
hsCRP<2 and Total Chol <5	93 (14.4)	10.61	1.00 (Ref)	1.00 (Ref)	1.00 (Ref)
hsCRP<2 and Total Chol ≥5	217 (13.4)	9.45	0.88 (0.69–1.13)	0.87 (0.68–1.11)	0.84 (0.62–1.12)
hsCRP ≥2 and Total Chol <5	106 (14.8)	11.71	1.11 (0.84–1.47)	1.06 (0.80–1.40)	1.08 (0.77–1.50)
hsCRP ≥2 and Total Chol ≥5	314 (13.6)	10.14	0.96 (0.76–1.20)	0.89 (0.70–1.12)	0.78 (0.59–1.05)
p-value			*0.75*	*0.63*	*0.19*
**Total coronary events & procedure**					
hsCRP<2 and Total Chol <5	189 (29.3)	22.67	1.00 (Ref)	1.00 (Ref)	1.00 (Ref)
hsCRP<2 and Total Chol ≥5	531 (32.8)	24.99	1.10 (0.93–1.30)	1.10 (0.94–1.30)	1.18 (0.96–1.46)
hsCRP ≥2 and Total Chol <5	263 (36.7)	31.51	1.42 (1.18–1.71)	1.40 (1.16–1.69)	1.36 (1.07–1.71)
hsCRP ≥2 and Total Chol ≥5	914 (39.5)	33.04	1.48 (1.26–1.73)	1.44 (1.23–1.68)	1.32 (1.08–1.61)
p-value			*<0.001*	*<0.001*	*0.01*
**Total CV events**					
hsCRP<2 and Total Chol <5	341 (53.0)	46.65	*1.00 (Ref)*	1.00 (Ref)	1.00 (Ref)
hsCRP<2 and Total Chol ≥5	843 (52.1)	43.75	0.93 (0.82–1.05)	0.93 (0.82–1.05)	0.95 (0.81–1.10)
hsCRP ≥2 and Total Chol <5	424 (59.1)	58.21	1.27 (1.10–1.47)	1.26 (1.09–1.45)	1.23 (1.03–1.46)
hsCRP ≥2 and Total Chol ≥5	1368 (59.1)	55.94	1.21 (1.08–1.36)	1.17 (1.04–1.32)	1.05 (0.91–1.22)
p-value			*<0.001*	*<0.001*	*0.12*
**All-cause mortality**					
hsCRP<2 and Total Chol <5	286 (44.4)	30.89	1.00 (Ref)	1.00 (Ref)	1.00 (Ref)
hsCRP<2 and Total Chol ≥5	669 (41.3)	27.91	0.89 (0.78–1.02)	0.90 (0.78–1.03)	0.84 (0.71–0.99)
hsCRP ≥2 and Total Chol <5	395 (55.1)	41.77	1.41 (1.21–1.64)	1.34 (1.15–1.56)	1.16 (0.96–1.39)
hsCRP ≥2 and Total Chol ≥5	1174 (50.7)	36.13	1.18 (1.04–1.34)	1.13 (0.99–1.29)	1.02 (1.07–1.19)
p-value			*<0.001*	*<0.001*	*0.20*

**Abbreviations:** HR: Hazard Ratio, CI: Confidence Interval CV: Cardiovascular, MI: Myocardial Infarction, CHD: Coronary Heart Diseases. hsCRP; High sensitivity C-reactive protein; Ref; Reference.

Reference (Ref) refers to the baseline group in a comparison, against which all other groups are evaluated when calculating the Hazard Ratio (HR).

**Model 1:** Adjusted for age, sex, socio-economic status (years of education) and ethnicity. **Model 2:** Model 1 adjusted further for a current smoker, body mass index, baseline SBP, creatinine, diabetes, history of vascular diseases (coronary, cerebral, peripheral), history of antihypertensive medication, and allocation to blood pressure-lowering and lipid-lowering.

Cox proportional hazards regression analysis was used to perform survival analysis, reporting hazard ratios (HR) with 95% confidence intervals (CI) and associated p-values.

a Per 1000 person-years.

**Table 5:** Risk of long-term cardiovascular events and mortality in relation to change of hsCRP and total Cholesterol after six months (n = 3717).

Outcomes	Events (%)	Rate^a^	Model 1, HR (95% CI)	Model 2, HR (95% CI)	Model 3, HR (95% CI)
**Non-fatal MI & fatal CHD**					
hsCRP decreased/Chol decreased	234 (13.3)	9.21	1.00 (Ref)	1.00 (Ref)	1.00 (Ref)
hsCRP decreased/Chol increased	140 (12.4)	8.80	0.93 (0.75–1.15)	0.96 (0.75–1.23)	0.98 (0.77–1.26)
hsCRP increased/Chol decreased	75 (17.8)	12.27	1.35 (1.04–1.75)	1.36 (0.97–1.88)	1.37 (0.98–1.91)
hsCRP increased/Chol increased	64 (15.7)	11.45	1.30 (0.98–1.71)	1.44 (1.03–2.01)	1.48 (1.05–2.08)
p-value			*0.02*	*0.02*	*0.02*
**Non-fatal & fatal Stroke**					
hsCRP decreased/Chol decreased	219 (12.4)	8.56	1.00 (Ref)	1.00 (Ref)	1.00 (Ref)
hsCRP decreased/Chol increased	158 (14.0)	9.71	1.12 (0.91–1.37)	1.11 (0.87–1.43)	0.90 (0.70–1.16)
hsCRP increased/Chol decreased	61 (14.5)	10.03	1.18 (0.89–1.56)	1.10 (0.78–1.56)	1.01 (0.71–1.44)
hsCRP increased/Chol increased	54 (13.3)	9.50	1.19 (0.88–1.60)	1.08 (0.75–1.54)	1.00 (0.69–1.45)
p-value			*0.14*	*0.65*	*0.96*
**Total coronary events & procedure**					
hsCRP decreased/Chol decreased	557 (31.6)	23.71	1.00 (Ref)	1.00 (Ref)	1.00 (Ref)
hsCRP decreased/Chol increased	371 (32.9)	25.49	1.07 (0.94–1.22)	1.07 (0.91–1.25)	1.09 (0.93–1.28)
hsCRP increased/Chol decreased	155 (36.8)	27.61	1.18 (0.99–1.41)	1.15 (0.92–1.45)	1.15 (0.92–1.45)
hsCRP increased/Chol increased	138 (33.9)	26.68	1.18 (0.98–1.43)	1.29 (1.02–1.61)	1.32 (1.04–1.66)
p-value			*0.03*	*0.03*	*0.02*
**Total CV events**					
hsCRP decreased/Chol decreased	921 (52.3)	43.71	1.00 (Ref)	1.00 (Ref)	1.00 (Ref)
hsCRP decreased/Chol increased	631 (55.9)	48.46	1.09 (0.99–1.21)	1.06 (0.94–1.19)	1.07 (0.95–1.21)
hsCRP increased/Chol decreased	244 (58.0)	49.02	1.17 (1.02–1.35)	1.12 (0.93–1.34)	1.07 (0.89–1.29)
hsCRP increased/Chol increased	215 (52.8)	46.67	1.14 (0.98–1.32)	1.21 (1.01–1.46)	1.19 (0.99–1.44)
p-value			*0.01*	*0.03*	*0.07*
**All-cause mortality**					
hsCRP decreased/Chol decreased	782 (44.4)	30.41	1.00 (Ref)	1.00 (Ref)	1.00 (Ref)
hsCRP decreased/Chol increased	544 (48.2)	33.51	1.08 (0.97–1.21)	1.17 (1.03–1.33)	1.22 (1.07–1.39)
hsCRP increased/Chol decreased	203 (48.2)	32.33	1.09 (0.93–1.27)	1.13 (0.93–1.37)	1.08 (0.89–1.31)
hsCRP increased/Chol increased	182 (44.7)	34.54	1.14 (0.97–1.34)	1.22 (1.00–1.49)	1.21 (0.99–1.48)
p-value			*0.07*	*0.03*	*0.05*

**Abbreviations:** HR: Hazard Ratio, CI: Confidence Interval CV: Cardiovascular, MI: Myocardial Infarction, CHD: Coronary Heart Diseases. Chol, Cholesterol; hsCRP; High sensitivity C-reactive protein; Ref; Reference.

Reference (Ref) refers to the baseline group in a comparison, against which all other groups are evaluated when calculating the Hazard Ratio (HR).

Groups were categorised based on changes in cholesterol and hsCRP levels from baseline to 6 months after the trial, depending on whether these levels increased or decreased during that period.

**Model 1:** Adjusted for age, sex, socio-economic status (years of education) and ethnicity. **Model 2:** Model 1 adjusted further for a current smoker, body mass index, baseline SBP, creatinine, diabetes, history of vascular diseases (coronary, cerebral, peripheral), history of antihypertensive medication, and allocation to blood pressure-lowering and lipid-lowering. **Model 3:** Model 3 adjusted further for baseline hsCRP and baseline total cholesterol.

Cox proportional hazards regression analysis was used to perform survival analysis, reporting hazard ratios (HR) with 95% confidence intervals (CI) and associated p-values.

a Per 1000 person-years.

**Fig. 2:**
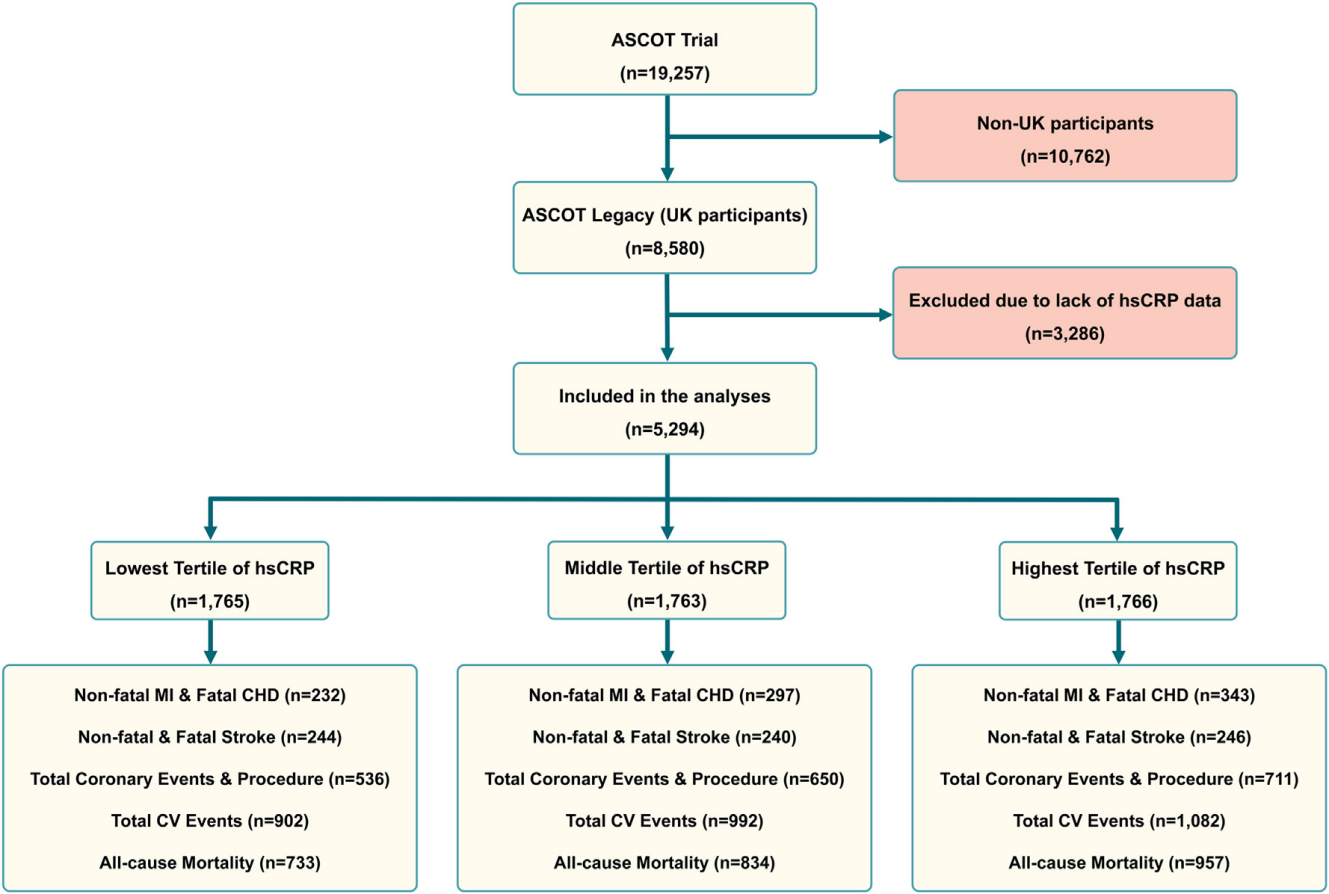
**Consort diagram of lipid-lowering (LL) arm of the ASCOT Legacy cohort.** CHD, coronary heart disease; CV, cardiovascular; hsCRP, high sensitivity C-reactive protein; MI, myocardial infarction.

**Table 6:** hsCRP risk model discrimination and reclassification (n = 1241).

Goodness of fit			Discrimination		Reclassification					
	LR	p-value (χ2 (df))	AUROC (95% CI)	p-value (diff) (DeLong test)	IDI	p-value (bootstrap method)	Continuous NRI	p-value (bootstrap method)	Categorical NRI	p-value (bootstrap method)
**Non-fatal MI & fatal CHD**										
Basic	Ref	Ref	0.7671 (0.7410–0.7926)	Ref	Ref		Ref		Ref	
hsCRP tertile	15.52 (16)	<0.001	0.7734 (0.7462–0.7988)	0.023	−0.40% (−0.90 to 0)	<0.001	9.68% (3.17–16.76)	<0.001	2.84% (−0.77 to 5.43)	0.042
**Non-fatal & fatal Stroke**										
Basic	Ref	Ref	0.7779 (0.7538–0.8016)	Ref	Ref		Ref		Ref	
hsCRP tertile	0.028 (16)	0.87	0.7779 (0.7540–0.8016)	0.42	0 (−0.10 to 0)	0.29	0.52% (−4.01 to 7.79)	0.43	0.19% (−0.1.88 to 2.32)	0.52
**Total coronary events & procedure**										
Basic	Ref	Ref	0.7624 (0.7389–0.7859)	Ref	Ref		Ref		Ref	
hsCRP tertile	20.42 (16)	<0.001	0.7663 (0.7417–0.7896)	0.10	−0.40% (−0.90 to 0)	<0.001	9.64% (3.23–16.54)	<0.001	0.40% (−0.31 to 1.19)	0.061
**Total CV events**										
Basic	Ref	Ref	0.7817 (0.7576–0.8055)	Ref	Ref		Ref		Ref	
hsCRP tertile	17.98 (16)	<0.001	0.7856 (0.7621–0.8093)	0.054	−0.40% (−0.90 to −0.10)	<0.001	9.66% (1.91–16.10)	<0.001	0.11% (−0.28 to 0.39)	0.31
**All-cause mortality**										
Basic	Ref	Ref	0.8133 (0.7976–0.8314)	Ref	Ref		Ref		Ref	
hsCRP tertile	21.50 (16)	<0.001	0.8154 (0.7994–0.8333)	0.071	−0.30% (−0.60 to 0)	0.10	8.96% (4.16–17.02)	<0.001	−0.04% (−1.08 to 1.59)	0.48

Model A (Basic): Adjusted for age, sex, socio-economic status (years of education), ethnicity, current smoker, body mass index, baseline SBP, creatinine, total cholesterol, diabetes, history of vascular diseases (coronary, cerebral, peripheral), history of antihypertensive medication, and allocation to blood pressure-lowering and lipid-lowering. Model B (hsCRP tertile): Model A + hsCRP (as a categorical variable, in tertiles).

hsCRP; high sensitivity C-reactive protein; IDI, Integrated Discrimination Improvement; LR, likelihood ratio; NRI, Net Reclassification Improvement; Ref, reference.

### Role of funders

The study funders had no role in study design, data collection, data analyses, interpretation, or writing of this report.

## Results

### Baseline characteristics

Of the 19,527 participants included in the LLA of the ASCOT population, 8580 were from the UK and are included in the ASCOT Legacy Study. Of these, 3286 participants did not have baseline hsCRP data available for analysis, leaving 5294 included in the final analysis (Fig. 2). Out of the 5294 study participants, 2524 were found to be alive, while 2770 had passed away. Among the surviving group, 2246 remained alive until January 31, 2019. Among them, 1012 participants experienced at least one cardiovascular event, while 1234 did not encounter any cardiovascular events. Additionally, 451 participants, who were reported alive during the study and had no cardiovascular events, were lost to follow-up. Among the deceased individuals, 860 individuals died due to cardiovascular events, while 1664 individuals passed away due to non-cardiovascular reasons, including cancer and respiratory infections. Non-fatal myocardial infarction and fatal CHD were the primary endpoint of the ASCOT, and the rest were secondary endpoints.

There was a relatively even divide when hsCRP was assessed dichotomously with hsCRP <2 mg/L (n = 2263) and ≥2 mg/L (n = 3031) subgroups. Of the low (<2 mg/L) hsCRP subgroup, 972 were allocated to placebo whilst 1006 were randomised to atorvastatin. In the high (≥2 mg/L) hsCRP subgroup, 1260 and 1251 were randomised to placebo and atorvastatin respectively. There were no substantial intra-group differences in cardiovascular outcomes by visualisation; however, there were appreciably more events in the high hsCRP group, which was assessed in further analyses.

Table 1 displays the baseline characteristics of the study population, as analysed between those allocated to placebo (n = 2232), atorvastatin (n = 2257) and those who were not randomised (n = 805). There were no substantial differences by visualisation in baseline characteristics between treatment allocation arms. The average age of participants was 65 years old and participants were 79–87% male, with approximately 25% being current smokers, 25% having diabetes and 17% who had prior coronary artery disease. Additionally, 90% were taking antihypertensive agents, with a mean systolic blood pressure of 162 mmHg and a body mass index of 29 kg/m^2^. Approximately 50% of each arm were randomised to either Atenolol or Amlodipine respectively.

### Relationship between baseline hsCRP in tertiles and outcomes

Next, the relationships between hsCRP in tertiles (with the lowest tertile as reference) and cardiovascular events were assessed (Table 2). This was performed in a crude analysis without correction; and with correction in Model 1 and also in Model 2.

The lowest tertile of hsCRP represented 0.01–1.51 mg/L, with median (IQR) of 0.88 (0.57–1.21) mg/L. The middle tertile was 1.52–3.71 mg/L, with median 2.41 (1.94–2.98) mg/L; whilst the highest tertile corresponded to 3.72–191.37 mg/L and median 6.41 (4.81–10.44) mg/L.

The highest tertile of hsCRP related to greater non-fatal MI and fatal CHD, the primary outcome of the original ASCOT, with HR 1.32 (95% CI 1.05–1.67, p = 0.019, [Cox proportional hazards regression]) relative to the lowest tertile. Similar relationships were seen with total coronary events and procedures, with HR 1.27 (95% CI 1.09–1.47, p = 0.0029, [Cox proportional hazards regression]), total cardiovascular events, with HR 1.22 (95% CI 1.08–1.37, p = 0.0009, [Cox proportional hazards regression]) and all-cause mortality with HR 1.27 (95% CI 1.10–1.42, p = 0.0011, [Cox proportional hazards regression]). There was insufficient evidence to suggest a true association between hsCRP levels and stroke/TIA. Unadjusted Kaplan–Meier curves were drawn for each composite endpoint (Fig. 1). The graded relationship from lowest to highest hsCRP tertile is clearly demonstrated for all endpoints, except stroke/TIA (all p < 0.0001, stroke/TIA, p = 0.48, p-values for Kaplan–Meier Curves has been reported using log-rank test). For all endpoints where a relationship exists (Fig. 1a, c–e), the event curves are continuing to diverge as follow-up time increases. Moreover, life tables provide detailed summary of events and survival data, showing the probability and the number of individuals at risk over specified intervals.

In sensitivity analyses, we repeated the analysis using competing risks regression models, which considered non-cardiovascular mortality as competing risks to cardiovascular mortality. The results were consistent with our aforementioned findings. Moreover, to ensure that there was no effect of being randomised in the ASCOT-LLA trial, the analysis was re-run, excluding the 805 participants who met the criteria for inclusion in the LLA, but were not randomised (n = 4489). Almost identical trends between hsCRP tertile and outcomes were observed (Supplementary Table S1).

### Relationship between baseline hsCRP in cut-off groups and outcomes

The relationship between hsCRP levels in subgroups (<2 mg/L [normal range], 2–4.9 mg/L, and ≥5 mg/L) were assessed. This resulted in three hsCRP groups: <2 mg/L (n = 2555; median [IQR]: 1.06 [0.65–1.46]), 2–4.9 mg/L (n = 1783; 3.07 [2.47–3.84]), and ≥5 mg/L (n = 1256; 8.15 [6.12–13.23]). The association between hsCRP groups and clinical endpoints was then evaluated using the <2 mg/L group as the reference (Table 3).

The hsCRP ≥5 mg/L subgroup showed weaker evidence of association with non-fatal MI and fatal CHD compared to the reference group (HR 1.22, 95% CI 0.96–1.57, p = 0.069, [Cox proportional hazards regression]). However, the highest hsCRP subgroup was associated with a greater incidence of total coronary events and procedures (HR 1.29, 95% CI 1.10–1.51, p < 0.0001, [Cox proportional hazards regression]), total cardiovascular events (HR 1.26, 95% CI 1.11–1.42, p < 0.0001, [Cox proportional hazards regression]), and all-cause mortality (HR 1.37, 95% CI 1.20–1.55, p < 0.0001, [Cox proportional hazards regression]). There was insufficient evidence to suggest a true association between hsCRP levels and stroke/TIA (HR 1.07, 95% CI 0.84–1.36, p = 0.70, [Cox proportional hazards regression]). Unadjusted and multivariable adjusted splines of the association between hsCRP and outcomes are shown in Supplementary Figure S1.

Unadjusted Kaplan–Meier curves were drawn for each composite endpoint (Supplementary Figure S1). The graded relationship from lowest to highest hsCRP subgroups is clearly demonstrated for all endpoints, except stroke/TIA (all p < 0.0001, stroke/TIA, p = 0.0.081, p-values for Kaplan–Meier Curves has been reported using log-rank test). For all endpoints where a relationship exists (Supplementary Figure S2a, c–e), the event curves are continuing to diverge as follow-up time increases. Moreover, Life tables provide a detailed summary of events and survival data, showing the probability and the number of individuals at risk over specified intervals.

As above, to ensure that there was no effect of being randomised in the ASCOT-LLA trial, the analysis was re-run, excluding the participants who met the criteria for inclusion in the LLA but were not randomised (n = 4489). Very similar trends between hsCRP subgroups and outcomes were observed (Supplementary Table S2).

### Interaction of baseline hsCRP with total cholesterol levels in predicting outcomes

Next, we examined the interaction between elevated total cholesterol (< or ≥5 mmol/L) and hsCRP (< or ≥2 mg/L) in relation to clinical events. The reference group comprised patients with total cholesterol <5 mmol/L and hsCRP <2 mg/L (see Table 4).

In a fully adjusted model, patients with both elevated total cholesterol and hsCRP experienced a higher incidence of non-fatal MI and fatal CHD (HR 1.27, 95% CI 0.95–1.71) compared to the reference group, with p = 0.029, [Cox proportional hazards regression]. Similarly, these patients also had a greater number of total coronary events and procedures (HR 1.32, 95% CI 1.08–1.61, p = 0.0079, [Cox proportional hazards regression]). This relationship was not significant in those with low hsCRP and raised cholesterol, whilst it was significant in those with raised hsCRP and low cholesterol. However, there was insufficient evidence to support a significant interaction between hsCRP and cholesterol concerning other outcomes.

This analysis was repeated after exclusion of non-randomised participants (Supplementary Table S3) with similar results.

### Relationship between temporal changes in hsCRP/total cholesterol at 6-months with outcomes

Any associations between change in hsCRP/total cholesterol levels with randomised therapy (atorvastatin or placebo) and clinical events were evaluated in 3717 patients who had 6-month follow-up blood results available (Table 5). Models 1 and 2 were used as previously, as well as Model 3, which incorporated additional adjustments for baseline hsCRP and total cholesterol. The reference group for this analysis was the group of patients where both hsCRP and total cholesterol were reduced at 6-months from baseline values, irrespective of the magnitude of changes in these parameters. The greatest adjusted risk (Model 3) was seen where both hsCRP and cholesterol increased at 6-months in those experiencing a non-fatal MI or fatal CHD (HR 1.48, 95% CI 1.05–2.08, p = 0.016, [Cox proportional hazards regression]), across groups. Similarly, elevated hsCRP and cholesterol with adjustment in Model 3 related to an increased number of coronary events and procedures (HR 1.32, 95% CI 1.04–1.66, p = 0.020, [Cox proportional hazards regression]). There was weaker evidence of the association between higher hsCRP and cholesterol with total cardiovascular events (HR 1.19, 95% CI 0.99–1.44, p = 0.072, [Cox proportional hazards regression]). There was insufficient evidence to establish a significant association between increased hsCRP and cholesterol with stroke/TIA (HR 1.00, 95% CI 0.69–1.45, p = 0.96, [Cox proportional hazards regression]).

### Improvement in outcome risk prediction with the inclusion of hsCRP

**Fig. 3:**
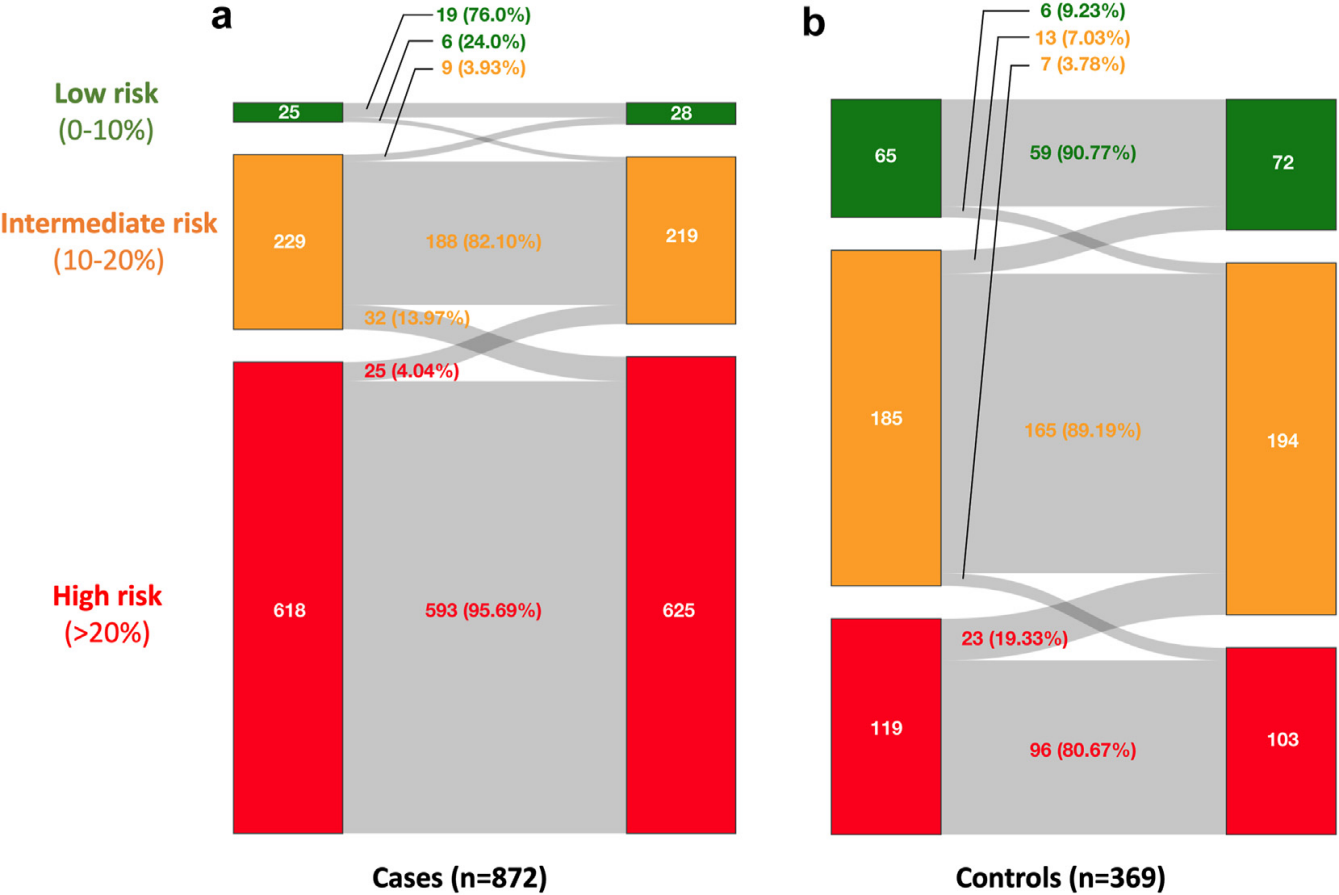
**Sankey diagram showing the reclassification of patients between risk categories for non-fatal MI or fatal CHD over 20 years, following the inclusion of hsCRP measured in tertiles into a basic model.** The reclassification has been reported separately for (a) cases (n = 872) and (b) controls (n = 369). The risk categories were 0–10% (low risk, green), 10–20% (intermediate risk, amber), and >20% (high risk, red). For both cases and controls, the left column represents the numbers of patients in each of the risk categories using the basic model, whilst the right column represents patient numbers in each risk category following the inclusion of hsCRP to the basic model. The basic model includes adjustment for age, sex, socio-economic status (years of education), ethnicity, current smoking status, body mass index, baseline SBP, creatinine, total cholesterol, diabetes, history of vascular diseases (coronary, cerebral, peripheral), history of antihypertensive medication, and allocation to blood pressure-lowering and lipid-lowering. Only participants with complete 20 year follow-up were included in this analysis.

Finally, we examined the added discriminatory power that the addition of hsCRP, as a categorical variable in tertiles (Table 6), and a continuous variable (Supplementary Table S4) has for the prediction of future cardiovascular endpoints at 20 years. Only participants with complete 20 year follow-up or who suffered a fatal event were included in this analysis (n = 1241). The IDI and NRI were assessed following the addition of hsCRP. There were significant improvements in both IDI and NRI with hsCRP for non-fatal MI and fatal CHD, total coronary events and procedures as well as total cardiovascular events. However, the largest improvement in NRI was seen with non-fatal MI and fatal CHD (9.68% for categorical hsCRP, 11.67% for continuous CRP, both p < 0.0001 [bootstrap method]). Accordingly, the probability of participants being reclassified, with the incorporation of hsCRP, into different risk categories (<10%, 10–20% and >20%) for the development of non-fatal MI or fatal CHD over 20 year follow-up, was then evaluated (Fig. 3—in tertiles, Supplementary Figure S3—continuous data). Among participants who experienced a non-fatal MI or fatal CHD over 20 years (cases), the proportion of participants who moved from a lower to a higher risk category increased after including hsCRP in the model. Among cases, the addition of hsCRP resulted in 38 individuals moving to a higher risk category (appropriate), indicating a more accurate classification of their risk, while 34 moved to a lower risk category (inappropriate), which might reflect an over-correction or misclassification. This resulted in a net change of 4 cases, corresponding to a net improvement of 0.46%. Among non-cases (controls), 36 individuals moved to a lower risk category (appropriate), and 13 moved to a higher category (inappropriate), resulting in a net improvement of 6.2%.

The AUROC increased with the inclusion of continuous hsCRP for all other outcomes. The AUROC for non-fatal MI and fatal CHD was 0.7671 without CRP (95% CI 0.7410–0.7926) increasing to 0.7715 (95% CI 0.7468–0.7952), with CRP p < 0.0001 [DeLong test]. There were weaker relationships with hsCRP in tertiles. The addition of baseline hsCRP to the basic model with all variables included, as previous, did not improve the prediction of future non-fatal and fatal stroke.

## Discussion

This study has a well-characterised patient population who were inherently stable and free from, but at risk of, cardiovascular disease at baseline, with long-term follow-up. Using this rare opportunity provided by the ASCOT Legacy study, we demonstrate the predictive power of a single measured biomarker at baseline in apparently clinically well individuals on 20 year cardiovascular outcomes.

Our key findings are that firstly, a modestly elevated baseline hsCRP in patients without overt cardiovascular disease predicted a range of important outcomes, with significant hazard ratios. Crucially, these clinical outcomes include events that are specific for CVD, as well as all-cause mortality. Secondly, hsCRP did not relate to the long-term incidence of stroke or TIA in this study. This may well reflect that stroke risk is less dependent on inflammatory pathways than coronary risk. The patients with elevated hsCRP at the baseline had increased risk of cardiovascular outcomes and mortality compared to those in the reference group over the many years of follow up, even when corrected to a comprehensive list of risk factors and confounders. This is remarkable, as one baseline measurement is seldom a good enough marker to predict risk at this magnitude over a long period of follow-up. The fact that the same measurement predicted all-cause mortality could also reflect the relevance of inflammation to many other pathologies, such as heart failure or chronic renal disease, for example.

Furthermore, a simultaneous increase in both hsCRP and cholesterol over 6 months was associated with a significantly higher risk of events at very long-term follow-up. However, a solitary increase in cholesterol levels at 6 months in the presence of a low hsCRP <2 mg/L did not convey a statistically significant increase. This suggests synergy in the dynamic relationship between the risk associated with cholesterol and inflammation. Accordingly, a recent study (2023) in over 30 000 patients with hyperlipidaemia enrolled in contemporary clinical trials who received intensive statin therapy, residual inflammatory risk evaluated by hsCRP was a stronger predictor of future cardiovascular events than LDL-cholesterol,^19^ suggesting the requirement for further adjunctive therapies to attenuate this risk, above and beyond lipid lowering. Furthermore, a recent substudy of the CLEAR-Outcomes trial (Cholesterol Lowering via Bempedoic Acid, an Adenosine triphosphate Citrate Lyase-Inhibiting Regimen Outcomes Trial) has again highlighted the importance of inflammation (assessed by hsCRP) in relationship to future cardiovascular events. In this study, Bempedoic Acid, principally considered an agent for hypercholesterolaemia, had similar efficacy for reducing hsCRP and LDL-cholesterol (∼21% at 6-months), yet baseline hsCRP was more strongly related to all cardiovascular outcomes than LDL-cholesterol over the 40.6 months of trial follow-up. Most strikingly, LDL-cholesterol was not related to cardiovascular mortality (HR 0.90, 95% CI 0.70–1.17), whilst hsCRP was associated (HR 2.00, 95% CI, 1.53–2.61).^20^ As a further example of the importance of inflammation in CVD, patients who achieve very low LDL levels with aggressive lipid reducing therapies, such as with proprotein convertase subtilisin/kexin type 9 serine protease (PCSK9) inhibitors, are still at substantial increased risk of further cardiovascular events. For example, in the randomised placebo-controlled clinical trial of evolocumab, ‘Further Cardiovascular Outcomes Research with PCSK9 Inhibition in Subjects with Elevated Risk’ (FOURIER), only a 2% absolute reduction in events was achieved versus placebo (12.6% versus 14.6% over 3 years).^21^^,^^22^ However, of course, these encouraging studies supporting the important association of inflammation with incident cardiovascular disease need to be tempered by the prior understanding that CRP is not causally related to CHD.^4^

Nonetheless, although hsCRP is considered to only be a correlative biomarker of cardiovascular disease, we have demonstrated in both acute and stable populations that very modest elevations relate to hard and important clinical endpoints.^13^ In the present study we calculated the added discriminatory capacity of a baseline hsCRP measurement on predicting clinical outcomes, over and above the cardiovascular risk factors included in the risk adjustment models, in particular for non-fatal MI and fatal CHD. The NRI shows the importance of using hsCRP to correctly allocate patients into risk categories and therefore into treatments.

Throughout all primary and secondary outcomes in this study there was a notable lack of a relationship between hsCRP and stroke events. Stroke is a heterogenous pathological entity, with different possible underlying pathologies leading to a similar outcome. These include plaque rupture from carotid arteries, or more commonly cardiac embolisation (e.g., atrial fibrillation) and of course haemorrhage, in the context of hypertension or vascular disease, as well as various predisposing haematological conditions. Therefore, as we have demonstrated previously in our case–control study in ASCOT,^23^ it is difficult to demonstrate a clear relationship between one biomarker with stroke outcomes. It may well be that plaque rupture leading to stroke in carotid disease has more in common with coronary disease, rather than embolisation.^24^

However, there is some data that has linked higher CRP levels with incidence of stroke, such as a pooled analysis by the Emerging Risk Factors Collaboration that reported an adjusted relative risk of stroke per 3-fold increase in CRP of 1.27 (95% CI 1.15–1.4).^25^ Similarly, a large genome-wide association study has confirmed the relationship of the Interleukin-6 receptor with all stroke events, except for those due to cardioembolism.^26^ Lastly, imaging biomarker studies, for example, using ^18^F-fluorodeoxyglucose positron emission tomography, have demonstrated an association between more metabolically active (inflammatory) plaques, stroke symptoms and markers of plaque instability.^27^^,^^28^ Another possible explanation for the lack of relationship between hsCRP and stroke outcomes in our cohort is due to the validity of medical health record stroke/TIA diagnosis, which may have a high degree of variability or misclassification, especially in primary care.^29^

Current clinical risk assessment tools utilise combined cardiovascular events as outcomes. Perhaps the differing significance of inflammation in coronary and cerebral events may mean that evaluation of individualised (coronary and stroke) outcomes may provide a more tailored risk assessment. Moreover, what constitutes a normal hsCRP level requires further clarification. In this study we have used cut-offs similar to the CANTOS trial in an additional analysis to provide non-tertile based data that may be informative to clinicians. However, when considering the utilisation of hsCRP for risk stratification for the development of future CVD, it will be important to consider hsCRP in tertiles, accompanied by other prognostically important biomarkers and clinical variables. Using artificially defined subgroup cut-offs of a continuous variable will not be effective in this regard or reflect inflammatory biology.

The recent results (2023) of the Prediction of Recurrent Events With ^18^F-Fluoride (PREFFIR) study, add further weight to the importance of inflammation in CVD. In this study, 704 patients post–MI were imaged with positron emission tomography using the ^18^F–NaF radiotracer and followed up for 4 years. Higher ^18^F–NaF signal independently related to increased MI and cardiac death, but not unplanned revascularisation or all-cause mortality.^30^ With the inclusion of anti-inflammatory therapy in CVD secondary prevention guidelines, the issue of uncertain mechanism (or even site) of drug action grows increasing importance. The anti-inflammatory therapy Colchine, used within 30 days of MI in COLCOT^11^ or in chronic coronary syndromes in LoDoCo-2^10^ has been shown to reduce major adverse cardiovascular events, yet whether it is acting on reducing atherosclerotic plaque inflammation is unclear.

Together, these studies clearly advocate for further consideration to include this simple measurement into risk matrices and to drive mechanistic studies to explore its relationships with various inflammatory pathways further. To support this concept, we believe that there is a mismatch between the effect sizes of certain anti-inflammatory therapies (e.g., 15% at 3.7 years with canakinumab in CANTOS^8^) and the larger expected risk reductions associated with elevated hsCRP reported in this study. More research is clearly required to explore this therapeutic targeting gap further. It may be that more continuous suppression of inflammation is needed, over a longer period than can be provided in the setting of a randomised controlled trial. To this end, the currently recruiting multi-centre placebo-controlled Zlitivekimab Cardiovascular Outcomes Study (ZEUS) (NCT05021835) will assess the role of IL-6 inhibition on cardiovascular events up to 4 years in 6200 patients at high atherosclerotic risk with chronic renal disease and elevated hsCRP (≥2 mg/L).

Thus, we would recommend that both hsCRP and cholesterol levels be measured at baseline prior to starting therapy to modulate cardiovascular risk factors (including statins, which have pleiotropic anti-inflammatory effects), to help guide the aggressiveness of preventative therapy.^31^

The primary limitation of the study lies in reporting outcomes as hazard ratios (HRs), the primary effect measure in this paper. While HRs are often interpreted practically as incidence rate ratios, their direct use for causal inference is not straightforward, even in the absence of unmeasured confounding, measurement error, and model misspecification. Assigning a causal interpretation to HRs carries inherent risks, primarily due to two key reasons: Firstly, HRs may vary over time, especially among individuals who do not experience events during the follow-up period, potentially leading to fluctuations in hazard magnitude. Consequently, relying solely on average HRs may yield incomplete insights, as period-specific HRs might exhibit time-varying patterns, influenced by inherent selection biases.^32^ Despite our relatively long-term follow-up duration, it's important to acknowledge that event risks could evolve over time within the same population. Extending the follow-up period further might reveal additional shifts in event risk dynamics among patients.

The second limitation of this study stems from its reliance on electronic health records for the clinical event outcomes. This may be particularly important for the TIA/stroke outcomes, where diagnostic accuracy may be highly variable, especially if diagnosed in primary care rather than by stroke specialist physicians. Moreover, we were not able to follow-up the entire ASCOT population, as identifiers were not available for Scandinavian, Irish cohorts, nor all UK participants. Therefore, imbalances in unmeasured confounders, given that randomisation was not stratified by country, are possible. Moreover, although measuring a one-off hsCRP level at baseline predicted outcomes 20 years later in this study, there is the possibility that there was an alternative reason for the hsCRP elevation at that point in time, such as an intercurrent infection, that may have been subsequently treated. As discussed, we are not claiming to have identified the cause for the minimal hsCRP elevations seen in this study but we provide supporting evidence for its importance.

The other limitation of our study is the cause-specific method employed our analysis. One considerable issue is the reliance on several strong assumptions, such as the independence of risk factors between censored and uncensored participants and the feasibility of practical interventions on competing events. In the context of this study, such assumptions may not always hold true. Additionally, the method assumes conditional exchangeability of censoring, meaning that the risk factors for those who are censored are the same as for those who are not, conditional on the measured variables. This assumption is challenging to meet, particularly in long-term studies where unmeasured confounders could significantly influence both the primary outcome and the competing events. For instance, lifestyle changes, adherence to medication, and other health interventions over the 20 year follow-up period could differentially affect the outcomes, leading to bias. Another limitation of this study is the potential for unmeasured confounding, which could influence the observed associations. Despite our efforts to adjust for known confounders, there remains a possibility that unmeasured variables could have impacted our results. Additionally, residual confounding due to measurement error in confounders is possible.

Other limitations of this study were the incomplete profile of hsCRP and cholesterol measurements after 6 months. A measure of CRP at baseline was an inclusion criterion for this sub-study, and thus there was no missing data for the main variables at the baseline, however, after 6 months, we have missing measurements of CRP. As a result, this analysis was restricted to participants with both baseline and 6-month measurements available. Although complete-case analysis is typically not the favoured approach in epidemiological research for managing missing data, it was chosen here due to the specific nature of the data and characteristics of the participants. Lastly, ASCOT utilised relatively low-intensity lipid lowering therapies by current practices, and a such the study findings may be less relevant in patient populations taking contemporary high-intensity and combination lipid-lowering therapies.

The strengths of this study are in its ability to demonstrate the predictive longevity of a single blood test at baseline in both risk stratification and reclassification at up to 20 years follow-up. We were pleased to show that hsCRP as a biomarker may become a selection tool for novel therapeutics that target inflammatory pathways, including anti-IL1B and anti-IL6 agents. We also hypothesise that this data will be important in the conception of future risk stratification guidelines that will go beyond focusing on conventional cardiovascular risk factors.

This analysis of ASCOT Legacy study demonstrates that higher baseline hsCRP levels independently predict cardiovascular events and all-cause mortality at very long-term follow-up in stable patients with hypertension. The addition of hsCRP into risk prediction models improved the discrimination and reclassification of individuals, particularly for non-fatal MI and fatal CHD, the primary outcome of the original ASCOT study. We recommend that these findings are considered when designing new risk algorithms for the development of future CVD.

## Contributors

Conceptualisation, Methodology—AH, SR, AK, PC, PS, RK.

Data curation—SR, CA.

Investigation—SR, AK.

Writing—original draft—AH, SR, AK, RK.

Writing—review and editing—AH, SR, AK, RK, PS, PW, NS.

Supervision—PS, RK.

All authors read and approved the final version of the manuscript. The original data relating to this paper was accessed and verified by SR, AK and PS.

## Data sharing statement

The data underlying this study were obtained from NHS Digital and, due to confidentiality agreements, cannot be shared publicly. For any queries, please email the corresponding author.

## Declaration of interests

Paul Welsh—research grants from Boehringer Ingelheim, AstraZeneca, Roche Diagnostics, Novartis. Honoraria from Novo Nordisk and Raisio.

Naveed Sattar—research grants from Boehringer Ingelheim, AstraZeneca, Roche Diagnostics, Novartis. Consulting fees for Abbott Laboratories, AbbVie, Amgen, AstraZeneca, Boehringer Ingelheim, Eli Lilly, Hanmi Pharmaceuticals, Janssen, Menarini-Ricerche, Novartis, Novo Nordisk, Pfizer, Roche Diagnostics, Sanofi. Honoraria from Abbott Laboratories, AbbVie, AstraZeneca, Boehringer Ingelheim, Eli Lilly, Janssen, Novo Nordisk, Sanofi.

Peter Sever—Pfizer supported the ASCOT study. Consulting fees and honoraria for Viatris.
