# Supplementary material for: The relationship of baseline high-sensitivity C-reactive protein with incident cardiovascular events and all-cause mortality over 20 years
Source: eBioMedicine. 2025 Jun 4;117:105786. doi: 10.1016/j.ebiom.2025.105786 (PMC12172296; doi:10.1016/j.ebiom.2025.105786)
Supplement: Supplementary Figures and Tables [file mmc1.docx]

**Supplementary Material**

**The relationship of baseline high-sensitivity C-reactive protein with incident cardiovascular events and all-cause mortality over 20-years**

**Authors:**

Adam Hartley*^1^

Somayeh Rostamian*^1^

Amit Kaura^1^

Paris Chrysostomou^1^

Paul Welsh^2^

Cono Ariti^1^

Naveed Sattar^2^

Peter Sever^$1^

Ramzi Khamis^$#1^

* Joint first author ^$^ Joint senior author

**Affiliations:**

1 – National Heart and Lung Institute, Imperial College London, UK

2 – Institute of Cardiovascular and Medical Sciences, University of Glasgow, Glasgow, UK

**Figure S1. Unadjusted and multivariable adjusted spline of association between hsCRP level and the hazard ratio for outcomes.**

|  |  |
| --- | --- |
|  |  |
|  |  |
|  |  |
|  |  |

*Adjusted for age, sex, socio-economic status (years of education), ethnicity, current smoker, body mass index, baseline SBP, creatinine, total cholesterol, diabetes, history of vascular diseases (coronary, cerebral, peripheral), history of antihypertensive medication, and allocation to blood pressure-lowering and lipid-lowering. The dotted lines around the spline curves represents the 95% confidence interval. The frequency distribution of hsCRP levels in patients is displayed at the bottom of each graph.*

**Figure S2. Kaplan Meier curves in relation to baseline hsCRP levels in subgroups and outcomes over 20-year follow-up in the ASCOT Legacy Cohort (n=5,294)**


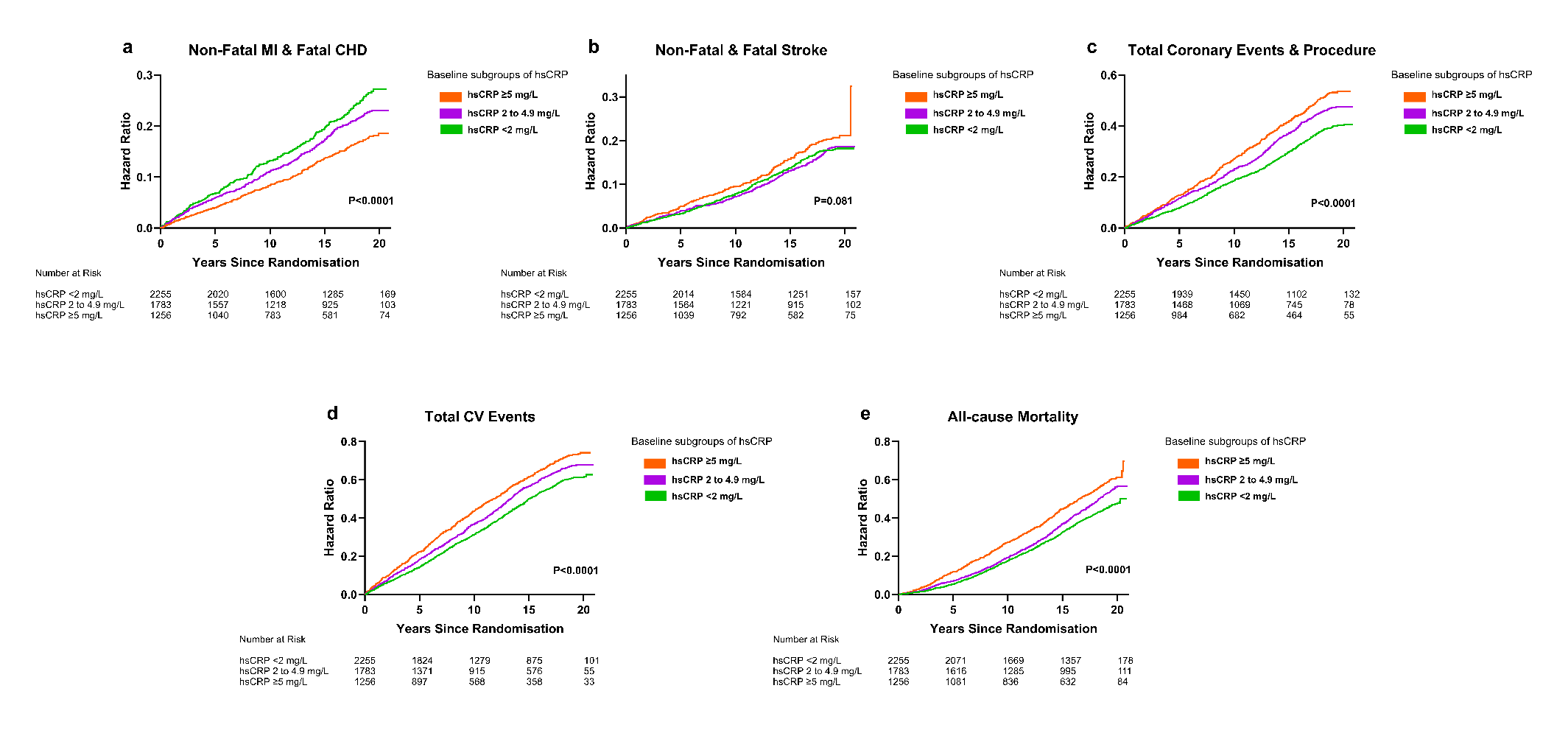


*(a)– Non-fatal MI and fatal CHD. (b) – Non-fatal and fatal stroke. (c) – Total coronary events and procedures (d) – Total cardiovascular events. (e) – All-cause mortality.*

***Abbreviations:*** *CHD: coronary heart disease; CV: cardiovascular; hsCRP: high sensitivity C-reactive protein.*

*[P-values reflect overall differences in survival probability between all hsCRP groups via the log-rank test (two-sided, α=0.05). The test evaluates whether the time-to-event distributions differ significantly across groups during follow-up.]*

*[Life tables has been provided detailed summary of events and survival data, showing the probability and the number of individuals at risk over specified intervals.]*

**Figure S3. Sankey diagram of the reclassification of patients between risk categories for non-fatal MI or fatal CHD over 20-years after inclusion of hsCRP (as a continuous variable) to a basic model**

*The reclassification has been reported separately for (a) cases and (b) controls. The risk categories were 0-10% (low risk), 10-20% (intermediate risk), and >20% (high risk). For both cases and controls, the left column represents the numbers of patients in each of the risk categories using the basic model, whilst the right column represents patient numbers in each risk category following the inclusion of hsCRP to the basic model. The basic model includes adjustment for age, sex, socio-economic status (years of education), ethnicity, current smoking status, body mass index, baseline SBP, creatinine, total cholesterol, diabetes, history of vascular diseases (coronary, cerebral, peripheral), history of antihypertensive medication, and allocation to blood pressure-lowering and lipid-lowering. Only participants with complete 20-year follow-up were included in this analysis.*

**Table S1. Association between baseline hsCRP in tertiles and outcomes in the ASCOT Legacy Cohort excluding non-randomized participants (n=4,489)**

| **Outcomes** | **Events (%)** | **Rate*** | **Crude,**  **HR (95%CI)** | **Model 1,**  **HR (95%CI)** | **Model 2,**  **HR (95%CI)** |
| --- | --- | --- | --- | --- | --- |
| **Non-fatal MI & Fatal CHD** |  |  |  |  |  |
| Lowest tertile | 173 (11.6) | 8.05 | 1.00 (Ref) | 1.00 (Ref) | 1.00 (Ref) |
| Middle tertile | 225 (15.0) | 10.81 | 1.35 (1.11-1.65) | 1.32 (1.08-1.61) | 1.32 (1.05-1.66) |
| Highest tertile | 247 (16.5) | 12.65 | 1.60 (1.31-1.94) | 1.59 (1.31-1.94) | 1.34 (1.06-1.70) |
| *P-value* |  |  | *<0.0001* | *<0.0001* | *0.017* |
| **Non-fatal & Fatal Stroke** | |  |  |  |  |
| Lowest tertile | 210 (14.0) | 9.99 | 1.00 (Ref) | 1.00 (Ref) | 1.00 (Ref) |
| Middle tertile | 194 (13.0) | 9.38 | 0.94 (0.77-1.14) | 0.89 (0.73-1.08) | 0.89 (0.71-1.12) |
| Highest tertile | 208 (13.9) | 10.76 | 1.09 (0.90-1.32) | 1.00 (0.83-1.22) | 0.99 (0.79-1.26) |
| *P-value* |  |  | *0.39* | *0.98* | *0.95* |
| **Total Coronary Events & Procedures** | | |  |  |  |
| Lowest tertile | 431 (28.8) | 21.66 | 1.00 (Ref) | 1.00 (Ref) | 1.00 (Ref) |
| Middle tertile | 519 (34.7) | 27.31 | 1.28 (1.13-1.45) | 1.24 (1.09-1.41) | 1.24 (1.06-1.44) |
| Highest tertile | 564 (37.7) | 31.84 | 1.51 (1.33-1.71) | 1.46 (1.29-1.66) | 1.27 (1.09-1.48) |
| *P-value* |  |  | *<0.0001* | *<0.0001* | *0.0029* |
| **Total CV Events** |  |  |  |  |  |
| Lowest tertile | 750 (50.1) | 42.16 | 1.00 (Ref) | 1.00 (Ref) | 1.00 (Ref) |
| Middle tertile | 810 (54.1) | 47.61 | 1.14 (1.03-1.26) | 1.09 (0.99-1.21) | 1.07 (0.95-1.20) |
| Highest tertile | 894 (59.7) | 58.08 | 1.41 (1.28-1.56) | 1.36 (1.24-1.50) | 1.20 (1.07-1.36) |
| *P-value* |  |  | *<0.0001* | *<0.0001* | *0.0019* |
| **All-cause Mortality** | |  |  |  |  |
| Lowest tertile | 619 (41.4) | 28.14 | 1.00 (Ref) | 1.00 (Ref) | 1.00 (Ref) |
| Middle tertile | 692 (46.3) | 32.09 | 1.15 (1.04-1.29) | 1.09 (0.98-1.22) | 1.02 (0.90-1.16) |
| Highest tertile | 816 (54.5) | 40.31 | 1.48 (1.34-1.65) | 1.41 (1.27-1.57) | 1.24 (1.10-1.41) |
| *P-value* |  |  | *<0.0001* | *<0.0001* | *<0.0001* |

**Abbreviations:** HR: Hazard Ratio, CI: Confidence Interval, CV: Cardiovascular, MI: Myocardial Infarction, CHD: Coronary Heart Diseases.

*Per 1000 person-year

**Model 1:** Adjusted for age, sex, socio-economic status (years of education) and ethnicity. **Model 2:** Model 1 adjusted further for a current smoker, body mass index, baseline SBP, creatinine, total cholesterol, diabetes, history of vascular diseases (coronary, cerebral, peripheral), history of antihypertensive medication, and allocation to blood pressure-lowering and lipid-lowering.

**hsCRP categories:**

Lowest tertile: n=1,496 à hsCRP: 0.01-1.47 [Median (IQR): 0.85 (0.55-1.16)]

Middle tertile: n=1,496 à hsCRP: 1.48-3.58 [Median (IQR): 2.33 (1.87-2.87)]

Highest tertile: n=1,497à hsCRP: 3.59-191.27 [Median (IQR): 6.24 (4.69-10.47)

**Table S2. Association between baseline hsCRP in subgroups and outcomes in the ASCOT Legacy Cohort excluding non-randomized participants (n=4,489)**

| **Outcomes** | **Events (%)** | **Rate*** | **Crude,**  **HR (95%CI)** | **Model 1,**  **HR (95%CI)** | **Model 2,**  **HR (95%CI)** |
| --- | --- | --- | --- | --- | --- |
| **Non-fatal MI & Fatal CHD** |  |  |  |  |  |
| hsCRP <2 | 248 (12.6) | 8.79 | 1.00 (Ref) | 1.00 (Ref) | 1.00 (Ref) |
| hsCRP 2 to 4.9 | 227 (15.2) | 9.7 | 1.27 (1.06-1.52) | 1.25 (1.04-1.50) | 1.24 (1.00-1.53) |
| hsCRP ≥5 | 170 (16.9) | 11.16 | 1.50 (1.24-1.83) | 1.51 (1.24-1.84) | 1.21 (1.11-1.72) |
| *P-value* |  |  | *<0.0001* | *<0.0001* | *0.065* |
| **Non-fatal & Fatal Stroke** | |  |  |  |  |
| hsCRP <2 | 271 (13.7) | 9.80 | 1.00 (Ref) | 1.00 (Ref) | 1.00 (Ref) |
| hsCRP 2 to 4.9 | 193 (13.0) | 9.49 | 0.97 (0.81-1.17) | 0.93 (0.77-1.12) | 0.94 (0.75-1.16) |
| hsCRP ≥5 | 148 (14.4) | 11.36 | 1.18 (0.96-1.44) | 1.09 (0.89-1.34) | 1.07 (0.84-1.36) |
| *P-value* |  |  | *0.18* | *0.53* | *0.70* |
| **Total Coronary Events & Procedures** | | |  |  |  |
| hsCRP <2 | 597 (30.3) | 22.77 | 1.00 (Ref) | 1.00 (Ref) | 1.00 (Ref) |
| hsCRP 2 to 4.9 | 519 (34.9) | 27.68 | 1.24 (1.10-1.39) | 1.21 (1.07-1.36) | 1.12 (0.97-1.28) |
| hsCRP ≥5 | 398 (38.7) | 33.46 | 1.52 (1.34-1.72) | 1.48 (1.30-1.69) | 1.29 (1.10-1.51) |
| *P-value* |  |  | *<0.0001* | *<0.0001* | *0.0014* |
| **Total CV Events** |  |  |  |  |  |
| hsCRP <2 | 1,006 (51.0) | 43.03 | 1.00 (Ref) | 1.00 (Ref) | 1.00 (Ref) |
| hsCRP 2 to 4.9 | 821 (55.2) | 49.54 | 1.17 (1.07-1.238) | 1.14 (1.04-1.25) | 1.09 (0.98-1.22) |
| hsCRP ≥5 | 627 (60.9) | 61.19 | 1.47 (1.33-1.62) | 1.43 (1.29-1.58) | 1.26 (1.11-1.42) |
| *P-value* |  |  | *<0.0001* | *<0.0001* | *0.0031* |
| **All-cause Mortality** | |  |  |  |  |
| hsCRP <2 | 833 (42.2) | 28.76 | 1.00 (Ref) | 1.00 (Ref) | 1.00 (Ref) |
| hsCRP 2 to 4.9 | 714 (48.0) | 33.64 | 1.19 (1.08-1.31) | *1.14 (1.03-1.26)* | *1.07 (1.57-1.97)* |
| hsCRP ≥5 | 580 (56.4) | 42.60 | 1.54 (1.39-1.71) | *1.50 (1.34-1.66)* | *1.37 (1.20-1.55)* |
| *P-value* |  |  | *<0.0001* | *<0.0001* | *<0.0001* |

**Abbreviations:** HR: Hazard Ratio, CI: Confidence Interval CV: Cardiovascular, MI: Myocardial Infarction, CHD: Coronary Heart Diseases.

*Per 1000 person-year

**Model 1:** Adjusted for age, sex, socio-economic status (years of education) and ethnicity. **Model 2:** Model 1 adjusted further for a current smoker, body mass index, baseline SBP, creatinine, total cholesterol, diabetes, history of vascular diseases (coronary, cerebral, peripheral), history of antihypertensive medication, and allocation to blood pressure-lowering and lipid-lowering.

**hsCRP categories:**

hsCRP <2 mg/L: n=1,972 à [Median (IQR): 1.03 (0.62-1.44)]

hsCRP 2 to 4.9 mg/L: n=1,488 à [Median (IQR): 3.06 (2.46-3.79)]

hsCRP ≥5 mg/L: n=1,029 à [Median (IQR): 8.34 (6.18-13.79)]

**Table S3. Risk of long-term cardiovascular events and mortality in relation to baseline hsCRP and total cholesterol excluding non-randomized participants (n=4,489)**

| **Outcomes** | **Events (%)** | **Rate*** | **Crude,**  **HR (95%CI)** | **Model 1,**  **HR (95%CI)** | **Model 2,**  **HR (95%CI)** |
| --- | --- | --- | --- | --- | --- |
| **Non-fatal MI & Fatal CHD** |  |  |  |  |  |
| hsCRP<2 and Total Chol <5 | 234 (13.3) | 8.94 | 1.00 (Ref) | 1.00 (Ref) | 1.00 (Ref) |
| hsCRP<2 and Total Chol ≥5 | 140 (12.4) | 8.68 | 0.97 (0.74-1.26) | 0.98 (0.75-1.28) | 1.01 (0.74-1.38) |
| hsCRP ≥2 and Total Chol <5 | 75 (17.8) | 11.63 | 1.32 (0.98-1.78) | 1.30 (0.97-1.75) | 1.17 (0.83-1.66) |
| hsCRP ≥2 and Total Chol ≥5 | 64 (15.7) | 11.93 | 1.34 (1.05-1.72) | 1.36 (1.06-1.74) | 1.27 (0.95-1.71) |
| *P-value* |  |  | *<0.0001* | *<0.0001* | *0.028* |
| **Non-fatal & Fatal Stroke** |  |  |  |  |  |
| hsCRP<2 and Total Chol <5 | 219 (12.4) | 10.38 | 1.00 (Ref) | 1.00 (Ref) | 1.00 (Ref) |
| hsCRP<2 and Total Chol ≥5 | 158 (14.0) | 9.50 | 0.91 (0.70-1.17) | 0.89 (0.69-1.15) | 0.84 (0.62-1.12) |
| hsCRP ≥2 and Total Chol <5 | 61 (14.5) | 11.89 | 1.16 (0.87-1.54) | 1.09 (0.82-1.46) | 1.08 (0.77-1.50) |
| hsCRP ≥2 and Total Chol ≥5 | 54 (13.3) | 9.67 | 0.93 (0.73-1.19) | 0.87 (0.68-1.11) | 0.78 (0.59-1.05) |
| *P-value* |  |  | *0.91* | *0.43* | *0.19* |
| **Total Coronary Events & Procedures** | |  |  |  |  |
| hsCRP<2 and Total Chol <5 | 557 (31.6) | 21.64 | 1.00 (Ref) | 1.00 (Ref) | 1.00 (Ref) |
| hsCRP<2 and Total Chol ≥5 | 371 (32.9) | 23.50 | 1.08 (0.91-1.29) | 1.09 (0.91-1.30) | 1.18 (0.96-1.46) |
| hsCRP ≥2 and Total Chol <5 | 155 (36.8) | 30.35 | 1.45 (1.19-1.76) | 1.42 (1.17-1.72) | 1.36 (1.07-1.71) |
| hsCRP ≥2 and Total Chol ≥5 | 138 (33.9) | 29.92 | 1.41 (1.19-1.66) | 1.37 (1.16-1.62) | 1.32 (1.08-1.61) |
| *P-value* |  |  | *<0.0001* | *<0.0001* | *0.0079* |
| **Total CV Events** |  |  |  |  |  |
| hsCRP<2 and Total Chol <5 | 921 (52.3) | 45.07 | 1.00 (Ref) | 1.00 (Ref) | 1.00 (Ref) |
| hsCRP<2 and Total Chol ≥5 | 631 (55.9) | 42.19 | 0.93 (0.81-1.06) | 0.93 (0.81-1.06) | 0.95 (0.81-1.10) |
| hsCRP ≥2 and Total Chol <5 | 244 (58.0) | 57.40 | 1.30 (1.13-1.51) | 1.29 (1.11-1.49) | 1.23 (1.03-1.46) |
| hsCRP ≥2 and Total Chol ≥5 | 215 (52.8) | 52.80 | 1.19 (1.05-1.34) | 1.15 (1.02-1.31) | 1.05 (0.91-1.22) |
| *P-value* |  |  | *<0.0001* | *<0.0001* | *0.12* |
| **All-cause Mortality** |  |  |  |  |  |
| hsCRP<2 and Total Chol <5 | 782 (44.4) | 30.17 | 1.00 (Ref) | 1.00 (Ref) | 1.00 (Ref) |
| hsCRP<2 and Total Chol ≥5 | 544 (48.2) | 28.19 | 0.93 (0.80-1.07) | 0.93 (0.80-1.07) | 0.84 (0.71-0.99) |
| hsCRP ≥2 and Total Chol <5 | 203 (48.2) | 41.73 | 1.44 (1.24-1.69) | 1.37 (1.17-1.60) | 1.16 (0.96-1.39) |
| hsCRP ≥2 and Total Chol ≥5 | 182 (44.7) | 35.53 | 1.19 (1.04-1.37) | 1.15 (1.00-1.32) | 0.98 (0.84-1.15) |
| *P-value* |  |  | *<0.0001* | *<0.0001* | *0.20* |

**Abbreviations:** HR: Hazard Ratio, CI: Confidence Interval CV: Cardiovascular, MI: Myocardial Infarction, CHD: Coronary Heart Diseases.

*Per 1000 person-year

**Model 1:** Adjusted for age, sex, socio-economic status (years of education) and ethnicity. **Model 2:** Model 1 adjusted further for a current smoker, body mass index, baseline SBP, creatinine, diabetes, history of vascular diseases (coronary, cerebral, peripheral), history of antihypertensive medication, and allocation to blood pressure-lowering and lipid-lowering

**Table S4. hsCRP risk model discrimination and reclassification (n=1,241)**

| **Goodness of fit** | | | **Discrimination** | |  |  | **Reclassification** | | | | | | |
| --- | --- | --- | --- | --- | --- | --- | --- | --- | --- | --- | --- | --- | --- |
|  | **LR** | **p-value (χ2 (df))** | **AUROC (95% CI)** | **p-value (diff) (DeLong test)** | **IDI** | | | **p-value (bootstrap method)** | **Continuous NRI** | **p-value (bootstrap method)** | **Categorical NRI** | **p-value (bootstrap method)** |  |
| **Non-fatal MI & Fatal CHD** | | |  |  |  | | |  |  |  |  |  |  |
| Basic |  | Ref | 0.7671  (0.7410 to 0.7926) | Ref |  | | | Ref |  | Ref |  | Ref |  |
| hsCRP | 7.30 (16) | 0.0071 | 0.7715  (0.7468 to 0.7952) | <0.0001 | -0.20%  (-0.50 to 0) | | | 0.10 | 11.67%  (3.97 to 20.49) | <0.0001 | 1.54%  (-0.35 to 3.05) | 0.051 |  |
| **Non-fatal & Fatal Stroke** | | |  |  |  | | |  |  |  |  |  |  |
| Basic |  | Ref | 0.7779  (0.7538 to 0.8016) | Ref |  | | | Ref |  | Ref |  | Ref |  |
| hsCRP | 0.13 (16) | 0.72 | 0.7782  (0.7538 to 0.8056) | 0.35 | 0  (-0.20 to 0.10) | | | 0.48 | 0.91%  (-6.92 to 11.12) | 0.49 | 0.073%  (-1.12 to 1.01) | 0.61 |  |
| **Total Coronary Events & Procedures** | | |  |  |  | | |  |  |  |  |  |  |
| Basic |  | Ref | 0.7624 (0.7389 to 0.7859) | Ref |  | | | Ref |  | Ref |  | Ref |  |
| hsCRP | 8.51 (16) | 0.0042 | 0.7658  (0.7395 to 0.7884) | 0.0074 | -0.30%  (-0.40 to -0.10) | | | <0.0001 | 9.65%  (2.10 to 17.26) | <0.0001 | 0.070%  (-0.22 to 0.41) | 0.34 |  |
| **Total CV Events** | | |  |  |  | | |  |  |  |  |  |  |
| Basic |  | Ref | 0.7817 (0.7576 to 0.8055) | Ref |  | | | Ref |  | Ref |  | Ref |  |
| hsCRP | 5.52 (16) | 0.021 | 0.7835  (0.7593 to 0.8089) | 0.022 | -0.20%  (-0.30 to 0) | | | <0.0001 | 8.31%  (4.08 to 15.46) | 0.022 | 0.056%  (-0.17 to 0.18) | 0.79 |  |
| **All-cause Mortality** | | |  |  |  | | |  |  |  |  |  |  |
| Basic |  | Ref | 0.8133 (0.7976 to 0.8314) | Ref |  | | | Ref |  | Ref |  | Ref |  |
| hsCRP | 21.19 (16) | <0.0001 | 0.8154 (0.7973 to 0.8337) | 0.023 | -0.30% (-0.50 to 0) | | | 0.10 | 8.10% (-2.30 to 17.73) | 0.083 | 0.03% (-0.68 to 0.55) | 0.54 |  |

**Abbreviations:** IDI, Integrated Discrimination Improvement; LR, likelihood ratio; NRI, Net Reclassification Improvement

**Model A (Basic):** Adjusted for age, sex, socio-economic status (years of education), ethnicity, current smoker, body mass index, baseline SBP, creatinine, total cholesterol, diabetes, history of vascular diseases (coronary, cerebral, peripheral), history of antihypertensive medication, and allocation to blood pressure-lowering and lipid-lowering. **Model B (hsCRP):** Model A + hsCRP (as a continuous variable). The term “Ref” refers to the reference model used for comparison between two models, with the associated p-value indicating the statistical significance of the comparison.
